# Genetic Diversity of the *Ralstonia solanacearum* Species Complex in the Southwest Indian Ocean Islands

**DOI:** 10.3389/fpls.2017.02139

**Published:** 2017-12-19

**Authors:** Noura Yahiaoui, Jean-Jacques Chéron, Santatra Ravelomanantsoa, Azali A. Hamza, Bobb Petrousse, Rajan Jeetah, Yasmina Jaufeerally-Fakim, Jérôme Félicité, Jacques Fillâtre, Bruno Hostachy, Fabien Guérin, Gilles Cellier, Philippe Prior, Stéphane Poussier

**Affiliations:** ^1^CIRAD, UMR Peuplements Végétaux et Bioagresseurs en Milieu Tropical, Saint-Pierre, France; ^2^Anses, National Plant Health Laboratory, Tropical Pests and Diseases Unit, Saint-Pierre, France; ^3^Université de la Réunion, UMR Peuplements Végétaux et Bioagresseurs en Milieu Tropical, Saint-Pierre, France; ^4^Centre National de la Recherche Appliquée au Développement Rural, Tananarive, Madagascar; ^5^Institut National de Recherche pour l'Agriculture, la Pêche et l'Environnement, Moroni, Comoros; ^6^Seychelles Agricultural Agency, Victoria, Seychelles; ^7^Food and Agricultural Research and Extension Institute, Curepipe, Mauritius; ^8^Department of Agriculture and Food Science, University of Mauritius, Réduit, Mauritius; ^9^Commission of Agriculture, Port-Mathurin, Mauritius; ^10^Association Réunionnaise pour la Modernisation de l'Economie Fruitière, Légumière et HORticole, Saint-Pierre, France; ^11^Institut National de la Recherche Agronomique, UMR Peuplements Végétaux et Bioagresseurs en Milieu Tropical, Saint-Pierre, France

**Keywords:** *Ralstonia solanacearum* species complex, Southwest Indian Ocean, genetic diversity, epidemiology, MLST, MLSA, clonal complex

## Abstract

Epidemiological surveillance of plant pathogens based on genotyping methods is mandatory to improve disease management strategies. In the Southwest Indian Ocean (SWIO) islands, bacterial wilt (BW) caused by the *Ralstonia solanacearum* species complex (RSSC) is hampering the production of many sustainable and cash crops. To thoroughly analyze the genetic diversity of the RSSC in the SWIO, we performed a wide sampling survey (in Comoros, Mauritius, Reunion, Rodrigues, and Seychelles) that yielded 1,704 isolates from 129 plots, mainly from solanaceous crops. Classification of the isolates to the four major RSSC phylogenetic groups, named phylotypes, showed that 87% were phylotype I, representing the most prevalent strain in each of the SWIO islands. Additionally, 9.7% were phylotype II, and 3.3% were phylotype III; however, these isolates were found only in Reunion. Phylotype IV (2 isolates), known to be restricted to Indonesia-Australia-Japan, was reported in Mauritius, representing the first report of this group in the SWIO. Partial endoglucanase (*egl*) sequencing, based on the selection of 145 isolates covering the geographic and host diversity in the SWIO (also including strains from Mayotte and Madagascar), revealed 14 sequevars with Reunion and Mauritius displaying the highest sequevar diversity. Through a multilocus sequence analysis (MLSA) scheme based on the partial sequencing of 6 housekeeping genes (*gdhA, gyrB, rplB, leuS, adk*, and *mutS*) and 1 virulence-associated gene (*egl*), we inferred the phylogenetic relationships between these 145 SWIO isolates and 90 worldwide RSSC reference strains. Phylotype I was the most recombinogenic, although recombination events were detected among all phylotypes. A multilocus sequence typing (MLST) scheme identified 29 sequence types (STs) with variable geographic distributions in the SWIO. The outstanding epidemiologic feature was STI-13 (sequevar I-31), which was overrepresented in the SWIO and obviously reflected a lineage strongly adapted to the SWIO environment. A goeBURST analysis identified eight clonal complexes (CCs) including SWIO isolates, four CCs being geographically restricted to the SWIO, and four CCs being widespread beyond the SWIO. This work, which highlights notable genetic links between African and SWIO strains, provides a basis for the epidemiological surveillance of RSSC and will contribute to BW management in the SWIO.

## Introduction

Epidemiological surveillance, defined as the continuous monitoring of diseases that are already present in a given region, was first developed in the field of human and animal health and then in plant health. All epidemiosurveillance networks involve data collection, analysis, and interpretation to provide crucial information for disease management (FAO, 2002). Multilocus sequence typing (MLST), the most popular genotyping method for epidemiological surveillance and outbreak investigations, was conceived in 1998 by clinical microbiologists who were working on *Neisseria meningitidis* (Maiden et al., 1998). Today, MLST and the multilocus sequence analysis (MLSA) technique (Gevers et al., 2005) are commonly used as portable and universal methods for bacterial identification, population genetic studies investigating the transmission pathways and spatiotemporal expansion of bacterial strains, and the determination of the phylogenetic relationships among bacterial strains (Urwin and Maiden, 2003; Hanage et al., 2005; Maiden, 2006).

Both MLSA and MLST examine nucleotide sequences of seven to eight loci that encode housekeeping genes that are carefully selected throughout the genome (Urwin and Maiden, 2003). These methods assess the contribution of mutation and recombination to the diversity and the evolutionary history of pathogens (Feil et al., 2004). MLST and MLSA have been successfully used to type and decipher the phylogenetic relationships among human and animal pathogenic bacteria, such as *Salmonella* spp. (Torpdahl et al., 2005; Roumagnac et al., 2006, 2007) and plant pathogens (Almeida et al., 2010), such as *Clavibacter* spp. (Jacques et al., 2012), *Xanthomonas* spp. (Ah-You et al., 2009; Fargier et al., 2011; Hamza et al., 2012), *Xylella fastidiosa* (Scally et al., 2005), and the *Ralstonia solanacearum* species complex (RSSC) (Wicker et al., 2012).

The RSSC (Gillings and Fahy, 1994), which is responsible for bacterial wilt (BW) disease, is considered one of the most destructive plant pathogenic bacteria (Mansfield et al., 2012). Common symptoms include the wilting of foliage, browning of vessels, and stunting of the plant (Hayward, 1994), ultimately leading to plant death. This soil-borne and xylem-invading plant pathogen causes high losses of many crops of economic importance from more than 50 botanical families (Hayward, 1994), including both monocots (e.g., *Musa* spp.) and dicots (e.g., *Solanum tuberosum* L.; He et al., 1983; Elphinstone, 2005). RSSC is distributed worldwide but BW epidemics are more intense in tropical and subtropical regions (Hayward, 1994). Cold-tolerant strains were introduced in Europe and North America in the 1990s (Janse, 1996; Elphinstone, 2005). BW disease is very difficult to control due to the ability of RSSC strains to survive freely for years in soil and water without a host plant (Kelman, 1956; Akiew, 1985; Caruso et al., 2005; Champoiseau et al., 2009) and the wide genetic diversity of RSSC strains. The heterogeneity of RSSC strains is mainly due to their capacity for natural transformation (Coupat et al., 2008) and recombination (Wicker et al., 2012). Effective control measures against BW are mostly based on prevention (epidemiological surveillance and elimination of inoculum sources) and the rational deployment of resistant varieties.

Historically, RSSCstrains that caused BW were classified into five races based on differences in host range (Buddenhagen et al., 1962) and six biovars based on differences in biochemical properties (Hayward, 1964). In 2005, Fegan and Prior proposed a new classification system based on a phylogenetic analysis of sequence data generated from the 16S-23S internal transcribed spacer (ITS) region. The strains that constitute the RSSC are distributed into four major phylogenetic groups, named phylotypes, according to their initial geographical origin: phylotype I from Asia, phylotype II from the Americas, phylotype III from Africa, and the Indian Ocean and phylotype IV from Australia-Japan-Indonesia (Fegan and Prior, 2005). More recently, the RSSC was split into three species, classifying phylotypes I and III as *R. pseudosolanacearum*, phylotype II as *R. solanacearum* and phylotype IV as *R. syzygii* (Safni et al., 2014; Prior et al., 2016). The phylotypes are further subdivided into sequevars based on nucleotide variations found in the partial sequencing of the endoglucanase (*egl*) gene (Fegan and Prior, 2005).

In the Southwest Indian Ocean (SWIO) islands (Comoros, Mauritius, Mayotte, Reunion, Rodrigues, and Seychelles), BW is regularly encountered and represents one of the most important factors that limits solanaceous crop production. The last devastating event caused by RSSC strains in the SWIO is still occurring in the potato production areas of Madagascar, seriously compromising food security (Ravelomanantsoa, 2016). This disastrous event clearly emphasizes the urgent need to implement a sentinel network across the SWIO to promptly detect and manage new outbreaks and predict potential introductions. Therefore, elucidating the genetic diversity of the RSSC strains and the dynamic structure of their populations appears to be a crucial step in the development of adapted BW control strategies in the SWIO area. Until now, only sporadic RSSC surveys have been conducted in the SWIO islands, and the genetic diversity of the prevalent RSSC strains on these islands has been scarcely documented.

In 1993, Girard et al. described the broad diversity of the RSSC strains on Reunion Island. They reported the presence of race 1/biovar 3 (now corresponding to phylotype I), race 3/biovar 2 (phylotype IIB—sequevar 1; IIB-1), and race 1/biovar 1 (phylotype III), with phylotype I as the most prevalent. Moreover, they reported that phylotype IIB-1 strains, responsible for potato brown rot, were exclusively present in the highlands (>1,000 m), but the other strains were present in low- and mid-altitude areas. In Mauritius, race 1/biovar 3 (i.e., phylotype I) strains were reported (Dookun et al., 2001). After 2005, Mauritius subsequently experienced three potato brown rot epidemics (2005, 2006, and 2008) caused by phylotype IIB-1 (Khoodoo et al., 2007, 2010). Moreover, the molecular characterization of scarce isolates from Comoros (2005), Mayotte (2006), and Seychelles (2007) led to the identification of only sequevar I-31 strains (unpublished data). A BW survey in 2012 in Mayotte generated a collection of 140 RSSC isolates with narrow genetic diversity, showing that sequevar I-31 was overrepresented (86%) (Chesneau et al., submitted).

To implement effective control measures against BW disease in the SWIO, such as the use of adapted, resistant cultivars, further extensive surveys of RSSC isolates need to be conducted. In this study, (i) the genetic diversity of RSSC isolates in the SWIO was assessed and (ii) compared with the genetic diversity of worldwide reference strains, and (iii) recombination events within the genes were revealed. Additionally, (iv) the structures of the RSSC populations in the SWIO area were evaluated and (v) compared with worldwide reference strains using MLSA/MLST, and (vi) we determined the effectiveness of the MLSA/MLST method.

This study revealed the presence of four phylotypes in the SWIO, with the first report of two phylotype IV isolates in Mauritius. The phylotypes' frequencies were highly variant among the islands, with Reunion displaying 3 phylotypes (I, II, and III), Mauritius with 2 phylotypes (I and IV) and only phylotype I in Mayotte, Comoros, Seychelles, and Rodrigues. Surprisingly, phylogenetic and genotyping approaches involving partial *egl* sequencing and MLSA/MLST revealed a very high prevalence of one genetic lineage (sequevar I-31; STI-13) throughout the SWIO islands that appeared to be closely linked to continental African RSSC strains.

## Materials and methods

### Collection of RSSC isolates in the SWIO

Wide sampling was conducted to cover crop production zones in each SWIO island (Comoros, Mauritius, Reunion, Rodrigues, and Seychelles). When possible, we collected ~30 random plants that showed BW disease-like symptoms in each plot (Hale et al., 2012). More than 2,000 stems on 129 plots were collected in the SWIO islands from November 2014 to February 2016. Plant samples were collected mainly from solanaceous crops (*Solanum lycopersicum, S. tuberosum, S. melongena*, and *Capsicum annuum)* and from *Pelargonium* cv. *rosé*, a traditional crop in Reunion Island that is grown for its very valuable essential oil, which is mainly used in the perfume industry and is known to be susceptible to BW (Girard et al., 1993). Surveys in the SWIO islands differed according to each specific topography and cultural habitat. The sampling elevation extended from sea level up to 1,706 m. For each sampled plant, a stem segment of 5–10 cm was cut. Stems were surface-disinfected with 70% ethanol and shredded for maceration in sterile distilled water to allow for the suspension of bacterial cells. Macerates (50 μl) were then individually streaked on tetrazolium chloride (TZC) agar medium (Kelman, 1954) or on modified semi-selective Granada and Sequeira medium (Poussier et al., 1999) for 48–72 h at 28°C, and one typical RSSC colony was purified for each sample on TZC medium. Finally, each isolate was phylotyped and stored at −80°C on Cryobank® microbeads (Microbank®, PRO-LAB DIAGNOSTICS, Neston, Wirral, U.K.) at the plant protection platform (Saint-Pierre, Reunion Island).

Ten collections of RSSC isolates were defined for our genetic studies (Table S1). The first collection, C1, was composed of 145 SWIO isolates (C1, *n* = 145) isolated during this study (Comoros, Mauritius, Reunion, Rodrigues, and Seychelles) or from previous surveys (Mayotte in 2012, Chesneau et al., submitted; Madagascar in 2012 and 2013; Ravelomanantsoa, 2016; Ravelomanantsoa et al., submitted) (Reunion, Maurice, Comoros, Seychelles from 1957 to 2007; unpublished data). This set of isolates was defined by selecting each isolate by date, geographical origin, plot, host of isolation, and phylotype. The second collection, C2, consisted of 91 worldwide reference strains covering the known RSSC phylogenetic diversity that were selected from a previous MLSA study (Wicker et al., 2012) (C2, *n* = 90). The third collection, C3, corresponded to C1+C2 (C3, *n* = 235). The SWIO collection (C1) was subdivided into seven sub-collections: 51 isolates from Reunion (C4, *n* = 51), 29 from Mauritius, (C5, *n* = 29), 25 from Madagascar (C6, *n* = 25), 18 from Comoros (C7, *n* = 18), 12 from Seychelles (C8, *n* = 12), 7 from Mayotte (C9, *n* = 7), and 3 from Rodrigues (C10, *n* = 3). The worldwide collection (C2) included RSSC strains from Africa (*n* = 23), America (*n* = 36), Asia (*n* = 28), Europe (*n* = 1), and Oceania (*n* = 2).

### Phylogenetic assignment

Two complementary approaches were used to determine the phylotypes of the SWIO RSSC isolates: multiplex PCR (Mmx-PCR) on the ITS region and a phylogenetic analysis based on the *egl* gene. The Mmx-PCR was conducted as previously described (Fegan and Prior, 2005).

A 1-μl loop of fresh colonies from a Petri dish was suspended in 200 μl of sterile HPLC water and was used as a template for PCR amplification. The Mmx-PCR used phylotype-specific primers to characterize all SWIO RSSC isolates. The identification of phylotypes I, II, III and IV was accomplished with four forward primers: Nmult 21:1F, Nmult 21:2F, Nmult 23:AF, and Nmult 22:InF, respectively, and Nmult 22:RR in the reverse direction. Each phylotype had an expected specific length of the amplified fragments [I: 144 base pairs (bp), II: 372 bp, III: 91 bp, and IV: 213 bp]. Mmx-PCR also included the RSSC-specific primer pair 759/760. The PCR conditions were as described by Fegan and Prior (2005): 25-μl reaction volumes containing 1X PCR buffer, 0.2 mM of each dNTP, 1.5 mM MgCl_2_, 2.4 μM of each phylotype-specific primer (Nmult 21:1F, Nmult 21:2F, Nmult 22:InF, and Nmult 22:RR), 7.2 μM of Nmult 23:AF, 1.6 μM of each species-specific primer, 0.625 U of GoTaq flexi DNA polymerase (Promega) and 2 μl of DNA suspension. The thermocycling conditions included an initial melting phase at 96°C for 5 min, followed by 30 cycles at 94°C for 15 s, 59°C for 30 s, and 72°C for 30 s and a final hold at 72°C for 10 min. Amplification was performed in a Verity technology thermocycler (Applied Biosystems thermal cyclers). The PCR products were analyzed by electrophoresis in 1.5% (110 V) agarose gels for 90 min and were revealed under UV light. A 100-bp DNA ladder and positive controls for each phylotype were added to each electrophoresis gel.

The phylogenetic assignment of SWIO RSSC isolates was also determined based on the partial nucleotide sequences of the endoglucanase (*egl)* gene. PCR amplification of a 750-bp region of the *egl* gene was performed using the Endo-F and Endo-R primers pair as previously described (Fegan et al., 1998; Poussier et al., 2000a). Fresh bacteria (2 μl) was amplified with 50-μl reaction volumes consisting of 10 μl of Colorless GoTaq Flexi Buffer (Promega), 3 μl of MgCl_2_ solution (25 mM), 1 μl of dNTP mix (10 mM each), 1.25 μl of a primer pair mix (10 μM each), 0.25 μl of GoTaq® G2 Flexi DNA Polymerase (PROMEGA), and 31.25 μl of sterile HPLC water. The steps of the protocol were carried out as follows: initial denaturation at 96°C for 9 min; 30 cycles of denaturation at 95°C for 1 min, 70°C for 1 min, and elongation at 72°C for 90 s; and a final extension at 72°C for 10 min. Then, 5 μl of the amplified PCR products was separated on a 1.5% agarose gel to visualize the amplification quality. PCR products were sent to Genewiz (Hope End Takeley, Essex CM22 6TA United Kingdom) for DNA double-stranded sequencing (forward and reverse). Forward and reverse chromatograms were edited using the Geneious v8.1.8 software package (Kearse et al., 2012). The determination of sequevars was assumed by *egl* sequence divergence values less than or equal to 1% (Fegan and Prior, 2005). After the assessment of the best-fit nucleotide substitution model, a phylogenetic tree based on *egl* sequences from SWIO and worldwide reference RSSC strains was reconstructed using PhyML v3.0 (Guindon et al., 2010).

### MLSA and MLST

Six housekeeping genes *(gdhA, gyrB, rplB, leuS, adk*, and *mutS*) and one virulence-associated gene (*egl*) were selected for MLSA and MLST studies as previously described (Wicker et al., 2012; Ravelomanantsoa et al., 2016). A set of primers (Table S2) were used to partially amplify the selected genes that were used for PCR and sequencing. The amplification protocol for the seven genes has previously been described (Wicker et al., 2012; Ravelomanantsoa et al., 2016). A total of 1,638 MLSA sequences were used in this study, including 657 that were retrieved from GenBank and 981 newly generated sequences that were deposited in GenBank under accession numbers KY862435 through KY863415. The accession numbers of the sequences are listed in Table S4. The consensus sequences were trimmed and aligned using the MUSCLE package (Edgar, 2004) in Geneious v8.1.8, and then the seven partial sequences *(gdhA, gyrB, rplB, leuS, adk, mutS*, and *egl*) were concatenated. For MLST, each unique sequence of a gene was assigned an allele number, and the combination of allele numbers for each isolate defined the sequence type (ST).

For single and concatenated gene sequences, the genetic diversities of the RSSC collections (C1 and C2) and of each phylotype were inferred by calculating the number of haplotypes (Hap), the number of polymorphic sites (S), the haplotype diversity (Hd), and the nucleotide diversity (Θπ) using DnaSP 5.0 (Librado and Rozas, 2009). Neutrality tests using the method of Tajima (1989) and Fu and Li (1993) (Tajima's D, Fu and Li's D^*^, and Fu's F^*^) were performed to elicit information about the evolutionary forces operating on each gene. Under conditions of neutrality, the expected value of these estimates is “0”; for the diversifying selection of genes, the expected value is positive; and under conditions of purifying selection, the expected value is negative. The Nei and Gojobori (1986) method was used to evaluate the synonymous/non-synonymous substitution (Ka/Ks) ratios with the MEGA 5.1 program. Under neutrality, the expected value of the Ka/Ks ratio is 1. For positively selected genes, the expected value of Ka/Ks is >1, and under purifying selection, the expected value of Ka/Ks is <1. The neighbor-net method implemented in the SplitsTree4 program was used to identify recombination events, calculated with the pairwise homoplasy index (PHI) test (Huson and Bryant, 2006). Recombination was estimated by the split decomposition of individual genes and with the concatenated dataset of 235 RSSC strains (collection C3), and the total recombination of each phylotype was estimated using the concatenated sequences of the seven genes. Split decomposition is a parsimony method that permits a tree-like network structure if conflicting phylogenetic signals are detected in the dataset (Huson and Bryant, 2006). The more reticulation there is in a network, the more conflicting signals exist in the sample, possibly due to the exchange of genetic material. The RDP4 program version 4.95 (Martin et al., 2010) provided an estimation of the recombination breakpoints and the mutation rate using seven nonparametric programs: RDP, GENECONV, BootScan, Chimaera, Maximum chi-square, SiScan, and 3Seq. Recombination events were accepted when they were detected with at least three of the seven detection methods.

The selection of the best-fit models of nucleotides was statistically calculated by jModelTest2 program version 2.1.9 (Posada, 2008) to construct phylogenetic trees for each gene and for recombination-free concatenated data. The topological congruence between the trees was tested by the Icong index based on maximum agreement subtrees (MAST). The Newick formats of the seven genes individually and of the concatenate were compared to each other at “http://max2.ese.u-psud.fr/icong/index.help.html” (de Vienne et al., 2007).

Based on the MLST data, the level of polymorphism was evaluated for each of the ten RSSC collections using the GenAlEx v6.5 software package (Peakall and Smouse, 2012) by computing the number of alleles per locus (Na), *Nei'*s marker of diversity index (H_E_). The allelic richness (A) estimates the genetic diversity in a population; it was calculated per locus and collection using the *allelic.richness* function in the “hierfstat” package (Goudet and Jombart, 2015). This calculation uses rarefaction to measure the number of alleles per locus in a random subsample of uniform size (*n* = 3) drawn from the collection. The discriminatory power of MLST was evaluated by calculating the Hunter-Gaston Discriminatory Index (HGDI) using the Discriminatory Power Calculator tool available at http://insilico.ehu.es/mini_tools/discriminatory_power/index.php (Hunter and Gaston, 1988; Hunter, 1990). This index measures the probability that two randomly sampled strains from a population will have different haplotypes. Loci with HGDI values < 0.3, 0.3–0.6, and >0.6 were considered poorly, moderately, and highly discriminative, respectively (Sola et al., 2003). MLST minimum spanning trees (MSTs) were built using the goeBURST full MST algorithm, which is based on the Euclidean and goeBURST distances between two STs (Kruskal, 1964), implemented in the PHYLOViZ v1.0 software package (Francisco et al., 2012). MSTs display the genetic diversity (number of haplotypes) within the collections of isolates and genetic relationships between haplotypes (STs). Clonal complexes (CCs) were defined as groups of genetically related haplotypes (STs) linked by a single-locus variant. A Chi-squared test and Fisher's exact test were used to analyze the associations between phylotypes and host plants on each island using R software version 0.99.903 (*n* > 15 isolates per host).

## Results

### A high prevalence of phylotype I in the SWIO

An extensive survey in the main crop production areas of the following SWIO islands allowed for the collection of 1704 RSSC isolates: Mauritius (*n* = 780, 35 plots), Reunion (*n* = 744, 63 plots), Seychelles (*n* = 68, 15 plots), Comoros (*n* = 61, 13 plots), and Rodrigues (*n* = 51, 3 plots; Table S1).

**Figure 1:**
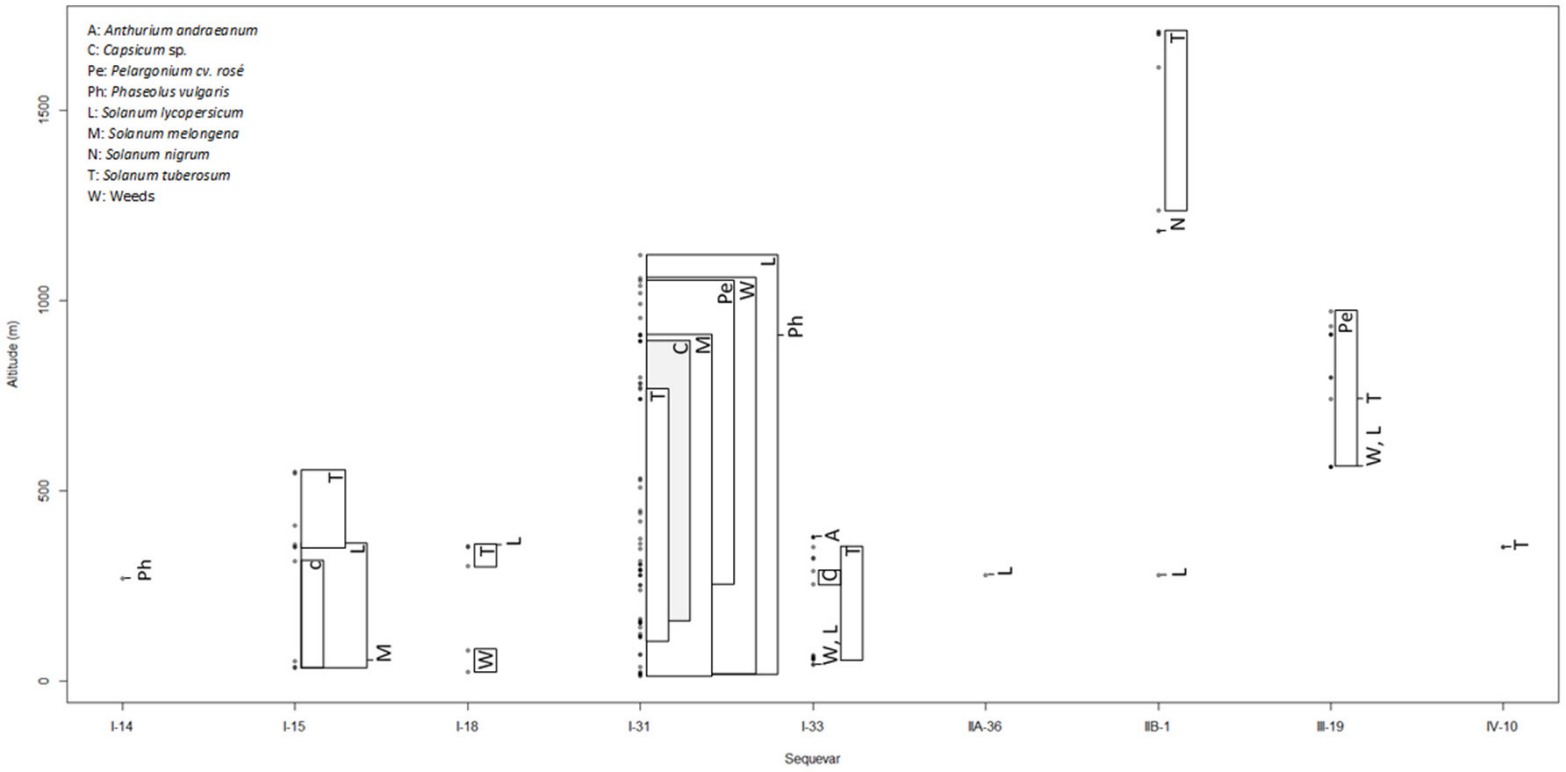
Distribution of sequevars of the RSSC depending on altitude and host of isolation in SWIO islands. For each sequevar, the dots correspond to the different altitudes of isolation. On the right of the dots, the letters indicate the host of isolation, and the rectangles mention the range of altitude of isolation for each plant.

In Reunion, phylotypes I, II, and III were identified with variable distributions and frequencies. Phylotype I isolates (*n* = 522, 43 plots), representing 70% of the collected isolates, were isolated throughout the island from sea level up to 1,120 m. Phylotype I isolates displayed a wider host range than phylotypes II and III and were collected more frequently from *S. lycopersicum, S. melongena*, and *Pelargonium* cv. *rosé* and more scarcely from *S. tuberosum, Capsicum* spp., *Phaseolus vulgaris*, and weeds (Figure 1 and Figure S1). Phylotype II isolates (*n* = 164 IIB-1, 8 plots, 22% of the collection) were isolated mainly from *S. tuberosum* and less frequently from *S. lycopersicum* (Figure 1 and Figure S1) in a restricted geographical area called “La Plaine des Cafres” in the highlands (from 1,184 to 1,706 m) and in two plots (at 280 and 1,243 m elevation) located in the south of this island. Only one isolate of IIA-36 was collected from *S. lycopersicum* at an altitude of 280 m. Phylotype III isolates (*n* = 57, 12 plots, 8% of the collection) were collected only in the western part of the island, mainly from *Pelargonium* cv. *rosé* but also from *S. tuberosum* and *S. lycopersicum* (Figure 1 and Figure S1). During the survey, we systematically revealed only one phylotype per plot from the 63 plots, except for two plots in which phylotypes I and II isolates were disclosed in a greenhouse and phylotypes I and III were found in an open field. Fisher's exact test revealed that phylotype II isolates were significantly described on *S. tuberosum* (*p* < 0.001), but on the other SWIO islands, no significant associations were observed between the phylotypes and the hosts.

In Mauritius, the survey led to the isolation of 780 isolates from 35 plots. All these isolates were phylotype I. Surprisingly, however, two isolates of phylotype IV were colocalized with phylotype I in the same potato plot. Mauritius isolates were most frequently collected from *S. tuberosum* and more scarcely from *S. lycopersicum, S. melongena, P. vulgaris*, and *Anthurium andreanum* (Figure S1).

In Comoros, Seychelles, and Rodrigues, fewer isolates (*n* = 61, 68, and 51 from 33 plots, respectively) were collected than in Reunion and Mauritius. On these islands, the RSSC genetic diversity was narrow since only phylotype I isolates were isolated, mostly from *S. lycopersicum* in Comoros, *S. melongena* in Seychelles and *S. lycopersicum* and *C. frutescens* in Rodrigues (Figure S1).

The prevalence of phylotype I in the SWIO islands was high. Indeed, of the 1704 RSSC isolates, 87% were phylotype I, 9.7% were phylotype II, 3.3% were phylotype III, and 2 strains were phylotype IV. Interestingly, we observed differences in the altitude of isolation among the phylotypes (Figure 1). Phylotype I varied from sea level up to 1,120 m, with an average of 366 m (Figure 1). Phylotypes II and III were isolated at comparatively higher altitudes, with an average of 1,245 m (from 280 to 1,706 m) and 786 m (from 564 to 973 m), respectively (Figure 1). The two phylotype IV strains were isolated at 354 m (Figure 1).

### A high prevalence of sequevar I-31 in the SWIO

Along with 90 worldwide reference strains (C2), 145 SWIO isolates (C1) were positioned within the global RSSC phylogenetic diversity by partial sequencing of the *egl* gene (Figure S2). In all the SWIO islands (including Mayotte and Madagascar), 14 sequevars were identified, with phylotypes I and II displaying the highest and lowest phylogenetic diversity, respectively. Unexpectedly, this analysis revealed the wide distribution and high prevalence of sequevar I-31, which was isolated from a wide host range (Figure 1 and Table S1) in all the SWIO islands, except Madagascar, representing 68% of SWIO phylotype I. Interestingly, among all the sequevars, sequevar I-31 presented the largest range of altitude, from sea level up to 1,120 m, while the other sequevars had more restricted ranges of altitude (<500; Figure 1). Phylogenetic diversity was broad in Reunion, where 6 sequevars were identified (I-13, I-31, I-33, IIA-36, IIB-1, and III-19), with a high prevalence of sequevar I-31 (94% of phylotype I). We also found wide phylogenetic diversity in Mauritius, with 6 sequevars (I-14, I-15, I-18, I-31, I-33, and IV-10) but a lower prevalence of sequevar I-31 (19% of phylotype I) than in Reunion. In Rodrigues, only isolates of sequevars I-31 and I-33 were disclosed. The Comoros (Anjouan and Mohéli) and Seychelles (Mahé and Praslin) archipelagos showed the lowest sequevar diversity in the SWIO, as only sequevar I-31 was revealed. Four sequevars reported in previous surveys in Madagascar (I-46, III-58, III-59, and III-60) and Mayotte (I-46) were not found in the other SWIO islands (Comoros, Mauritius, Reunion, Rodrigues, and Seychelles). No significant associations were found between the sequevars and the host plants, except between sequevar IIB-1 and *S. tuberosum*.

### A similar trend of RSSC genetic diversity in the SWIO and worldwide

Genetic diversity analyses were performed on the partial sequences of seven genes (*gdhA, gyrB, rplB, leuS, adk, mutS*, and *egl*). The total length of the concatenated sequences was 4,245 bp, corresponding to 603 bp for *gdhA*, 391 bp for *gyrB*, 654 bp for *rplB*, 723 bp for *leuS*, 468 bp for *adk*, 693 bp for *mutS*, and 713 bp for *egl* (Table 1 and Table S3). Synonymous substitutions (Ks) were more common than nonsynonymous substitutions (Ka) in the seven genes (from 3.4 to 23.2 and from 3.6 to 26.3 times more common in the SWIO and worldwide, respectively). All Ka/Ks values were in the range 0–1, indicating that the seven genes were under purifying selection and were therefore suitable for population studies (Maiden, 2006). The Tajima's D values were negative for the majority of the genes, meaning that the genes were under purifying selection, although none of the values were significant. All loci were polymorphic in the SWIO (C1) and worldwide (C2) RSSC collections, except for the two Mauritian phylotype IV isolates and *leuS* from the SWIO phylotype III isolates. A similar trend of genetic diversity was observed in both collections, although as expected less diversity was found in the SWIO collection than in the worldwide collection. The number of haplotypes, the number of polymorphic sites and the haplotype diversity were higher for C2 (Hap = 28, S = 397, Hd = 0.998) than C1 (Hap = 84, S = 660, Hd = 0.758). When considering C1, nucleotide diversity (Θπ) was 1.6% and was similar across genes, except for *egl*, which was more variable (Θπ = 3.5%), but it was 3.2% for C2 (*egl* remained the most variable, Θπ = 6%). When we focused on the number of polymorphic sites, *adk* was the least diverse gene (S = 32 for C1 and C2), and *egl* was the most diverse gene (S = 121 for C1 and 193 for C2). Among the phylotypes (except for phylotype IV, for which we had only two Mauritius strains) in the SWIO and the worldwide collections, phylotypes II and III appeared as the most diverse (Θπ = 0.3 and 0.4% for C1, and 1.5 and 1.2% for C2), and phylotype I appeared as the least diverse (Θπ = 0.2% for C1 and 0.6% for C2).

**Table 1:** Genetic properties of the loci used in MLSA for the SWIO (C1) and the worldwide (C2) RSSC collections.

**Collection (number of strains)**	**Locus**	**Length (bp)**	**Hap**	**S**	**Hd**	**G+C**	***Θπ***	**Tajima's D**	**Fu & Li's D**	**Fu's F**	**Ka/Ks**
**C1 (*****n*** = **145)**	*gdhA*	603	8	39	0.521	0.664	0.008	−0.909	0.963	7.280	0.174
	*gyrB*	391	15	38	0.731	0.645	0.022	0.564	1.258	5.950	0.077
	*rplB*	654	10	62	0.539	0.647	0.012	−0.984	0.331	10.421	0.043
	*leuS*	723	7	42	0.575	0.647	0.010	−0.289	1.064	13.288	0.094
	*adK*	468	14	32	0.732	0.647	0.014	−0.143	1.503	3.967	0.085
	*mutS*	693	9	62	0.620	0.715	0.014	−0.548	0.566	15.467	0.122
	*egl*	713	14	121	0.738	0.686	0.035	0.106	1.523	30.295	0.292
	Concatenate	4,245	28	397	0.758	0.667	0.016	−0.304	1.270	48.105	0.127
**C2 (*****n*** = **90)**	*gdhA*	603	36	84	0.943	0.660	0.023	−0.668	−1.483	−3.122	0.147
	*gyrB*	391	60	60	0.987	0.649	0.028	−0.517	−1.288	−37.527	0.120
	*rplB*	654	35	92	0.942	0.646	0.034	0.371	−0.101	0.677	0.038
	*leuS*	723	42	91	0.944	0.645	0.023	−0.458	−1.179	−4.991	0.105
	*adK*	468	53	32	0.976	0.651	0.020	0.764	0.155	−30.306	0.109
	*mutS*	693	45	109	0.955	0.711	0.032	−0.431	−2.217	−4.027	0.098
	*egl*	713	63	193	0.988	0.680	0.060	0.068	−0.080	−7.357	0.275
	Concatenate	4,245	84	660	0.998	0.665	0.032	−0.164	−0.963	−10.831	0.128

The number of haplotypes (Hap), the number of polymorphic sites (S), the haplotype diversity (Hd), and the nucleotide diversity (Θπ) were calculated using DnaSP 5.0 (Librado and Rozas, 2009). Neutrality tests using the method of Tajima (1989) and Fu and Li (1993) (Tajima's D, Fu and Li's D*, and Fu's F*) were performed to elicit information about the evolutionary forces operating on each gene. The Nei and Gojobori (1986) method was used to evaluate the synonymous/non-synonymous substitution (Ka/Ks) ratios with the MEGA 5.1 program. Significance: ^*^P < 0.05; ^**^P < 0.02; ^***^P < 0.001.

### Recombination is ubiquitous within the RSSC, with phylotype I as the most recombinogenic

We constructed phylogenetic networks using the neighbor-net algorithm to highlight conflicting signals in the gene sequence dataset, which would suggest the occurrence of recombination events within or across genes in RSSC strains (the C3 collection, including the representative SWIO and worldwide strains) (Figure S2). Except for *adk* and *mutS*, which showed less reticulate structures than other genes, all split graphs revealed many parallelograms, suggesting that recombination should have contributed to genetic evolution within the RSSC. From the concatenated sequence set, the PHI test confirmed the presence of significant recombination in each phylotype (Table 2; *p* < 0.005). From each gene separately, significant recombination was revealed in *mutS* for phylotype II and in *egl* for phylotypes II, III, and IV (Table 2).

**Table 2:** Recombination as assessed by the pairwise homoplasy index (PHI) test F_w_ determined using Splitstree (Huson and Bryant, 2006).

**Locus**	**Phylotype I (*****n*** = **130)**			**Phylotype II (*****n*** = **54)**			**Phylotype III (*****n*** = **38)**			**Phylotype IV (*****n*** = **13)**			**Concatenate (*****n*** = **235)**		
	**IS^a^**	**Mean F_w_**	***P*-value**	**IS**	**Mean F_w_**	***P*-value**	**IS**	**Mean F_w_**	***P*-value**	**IS**	**Mean F_w_**	***P*-value**	**IS**	**Mean F_w_**	***P*-value**
*gdhA*	5	0	1	16	0.100	7.379E-02	14	0.253	2.123E-01	22	0.173	1.312E-01	61	0.154	6.803E-02
*gyrB*	26	0.382	5.183E-01	19	0.351	5.502E-02	12	0.167	2.438E-01	22	0.290	2.618E-01	47	0.297	1.359E-01
*rplB*	5	0	1	25	0.117	2.880E-02	12	0.227	5.651E-01	34	0.291	4.630E-02	78	0.204	3.434E-02^*^
*leuS*	2	0	1	26	0.031	7.807E-02	9	0.139	1.100E-01	22	0.117	9.402E-01	64	0.127	6.592E-01
*adk*	15	0.200	5.062E-02	18	0.379	1.810E-01	11	0.073	3.317E-01	16	0.083	3.537E-01	32	0.268	5.336E-01
*mutS*	7	0.095	1.932E-01	29	0.113	3.806E-06^*^	22	0.255	1.796E-01	24	0	0	83	0.115	3.333E-01
*egl*	18	0.222	3.698E-01	57	0.104	1.959E-05^*^	37	0.264	1.008E-06^*^	49	0.196	2.837E-07^*^	172	0.149	3.849E-08^*^
Concatenate	78	0.298	2.645E-04^*^	190	0.217	0^*^	117	0.348	1.721E-15^*^	189	0.434	0^*^	537	0.2918369	0^*^

Analyses were performed on each phylotype of the RSSC and on each locus of the MLSA scheme. IS, Informative site;

* *Statistically significant evidence for recombination*.

Using RDP4, 13 individual recombination events were detected in the concatenated gene sequences set (Table 3). Only one gene, *gdhA*, was recombination-free. The six other genes showed one to four recombination events, *egl* being the most recombinogenic. These recombination events were identified in all phylotypes confirming that recombination is ubiquitous within the RSSC. Phylotype I appeared as the most recombinogenic since recombination affected 137, 2, 3, and 11 isolates belonging to phylotypes I–IV, respectively. Three recombination events (number 2, 5, and 12) were detected in SWIO isolates, two from phylotype I, and one from phylotype IV. Recombinant sequences originated primarily from phylotypes I and IV.

**Table 3:** Recombination events detected within RSSC.

**Recombination event number**	**Region**	**Gene**	**Recombinant sequence**	**Sequevar**	**Country**	**Major parental sequence^a^**	**Sequevar**	**Country**	**Minor parental sequence^b^**	**Sequevar**	**Country**	**Detection methods**						
												**RDP**	**GENECONV**	**BootScan**	**MaxChi**	**Chimaera**	**SiScan**	**3Seq**
**1**	3533–4245	*egl*	RUN0064	IV-9	Indonesia	RUN0608	I-13	Reunion	RUN1360	IV-9	Indonesia	4.393*10^−8^	1.615*10^−4^	NS^d^	6.868*10^−9^	NS	2.187*10^−28^	1.369*10^−30^
**2**	2840–3532	*mutS*	RUN1431	I-31	Mayotte	RUN1528	I-14	Guatemala	RUN0160	IIB-1	Reunion	2.085*10^−19^	3.992*10^−18^	2.082*10^−19^	6.931*10^−8^	3.599*10^−8^	8.241*10^−8^	1.388*10^−21^
**3**	1649–4245	*leuS to egl*	RUN1359	IV-na	Indonesia	RUN0083	IV-10	Indonesia	RUN1528	I-14	Guatemala	1.970*10^−10^	1.194*10^−7^	2.499*10^−9^	5.417*10^−7^	5.752*10^−12^	4.906*10^−10^	2.928*10^−20^
**4**	995–1648	*rplB*	RUN0081	IIB-28	Brazil	RUN0299	IIB-27	Brazil	RUN0083	IV-10	Indonesia	2.534*10^−18^	9.303*10^−17^	2.215*10^−18^	8.578*10^−6^	4.760*10^−7^	8.149*10^−11^	5.318*10^−12^
**5**	3533–4245	*egl*	RUN4847^1^	IV-10	Mauritius	RUN0071	IV-8	Japan	RUN0014	IV-11	Australia	NS	NS	NS	3.786*10^−3^	1.986*10^−3^	8.663*10^−8^	7.907*10^−11^
**6**	995–1648	*rplB*	RUN0076	III-20	Zimbabwe	RUN0075	III-22	Zimbabwe	RUN0160	IIB-1	Reunion	3.520*10^−12^	1.305*10^−10^	1.513*10^−12^	7.892*10^−8^	2.607*10^−7^	1.232*10^−8^	5.380*10^−15^
**7**	2372–2839	*adk*	RUN1530	IIA-6	Guatemala	RUN0454	IIA-6	Venezuela	Unknown^c^	Unknown	Unknown	NS	1.858*10^−14^	1.127*10^−15^	1.560*10^−3^	1.554*10^−3^	2.182*10^−4^	3.861*10^−9^
**8**	2372–2839	*adk*	RUN1528	I-14	Guatemala	RUN0054	I-18	French Guiana	RUN1526	IIA-7	USA	6.029*10^−10^	6.860*10^−9^	5.603*10^−10^	2.783*10^−2^	2.672*10^−2^	1.932*10^−4^	7.962*10^−6^
**9**	604–994	*gyrB*	RUN0089	IV-9	Indonesia	RUN1357	IV-9	Indonesia	Unknown	Unknown	Unknown	4.068*10^−9^	1.842*10^−5^	3.840*10^−9^	4.158*10^−2^	NS	NS	6.034*10^−4^
**10**	604–994	*gyrB*	RUN0159^2^	I-15	Taiwan	RUN5456	I-33	Rodrigues	RUN0297	IIB-5	Trinidad	1.448*10^−8^	1.441*10^−5^	3.710*10^−7^	1.431*10^−2^	1.061*10^−2^	4.623*10^−3^	2.986*10^−5^
**11**	2372–2839	*adk*	RUN1359^3^	IV-na	Indonesia	RUN0157	I-15	Taiwan	RUN0454	IIA-6	Venezuela	NS	1.652*10^−7^	1.417*10^−5^	4.236*10^−2^	4.041*10^−2^	1.932*10^−4^	9.866*10^−6^
**12**	3533–3920	*egl*	RUN1431^4^	I-31	Mayotte	RUN0364	III-42	Guinea	RUN1357	IV-9	Indonesia	9.515*10^−7^	4.627*10^−6^	>1.0	8.666*10^−1^	2.079*10^−3^	6.254*10^−1^	4.837*10^−6^
**13**	604–994	*gyrB*	RUN0075^5^	III-22	Zimbabwe	RUN5456	I-33	Rodrigues	RUN0150	IIA-41	Cameroon	1.110*10^−4^	1.169*10^−4^	7.100*10^−5^	4.092*10^−2^	3.318*10^−2^	NS	3.194*10^−3^

*Columns indicate the recombinant strain, corresponding sequevar and country, major and minor parents and recombinogenic area identified with a least three of the seven detection methods. Gene boundaries in the concatenated sequence are as follows: gdhA: 1–603; gyrB: 604–995; rplB: 995–1648; leuS: 1649–2371; adk: 2372–2839; mutS: 2840–3532, egl: 3533–4245. Sequences are identified by their RUN number*.

1 *RUN4606 (IV-10, Mauritius); RUN1361 (IV-11, Indonesia); RUN0062 (IV-10, Indonesia); RUN0063 (IV-10, Indonesia); RUN0083 (IV-10, Indonesia)*.

2 *RUN0157 (I-15, Taiwan); RUN0047 (I-45, Philippines); RUN0337 (I-48, China)*.

3 *RUN0339 (I-44, China)*.

4 *131 sequences (130 are phylotype I: all phylotype I in this study, only RUN1359 is IV-na)*.

5 *RUN0145 (III-29, Cameroon)*.

a *Major Parent, Parent contributing the larger fraction of recombinant sequence*.

b *Minor Parent, Parent contributing the smaller fraction of recombinant sequence*.

c *Unknown, A missing parental sequence. Only one parent and a recombinant need to be in the alignment for a recombination event to be detectable*.

d *NS, No significant p-value was recorded for this recombination event using this method*.

**Figure 2:**
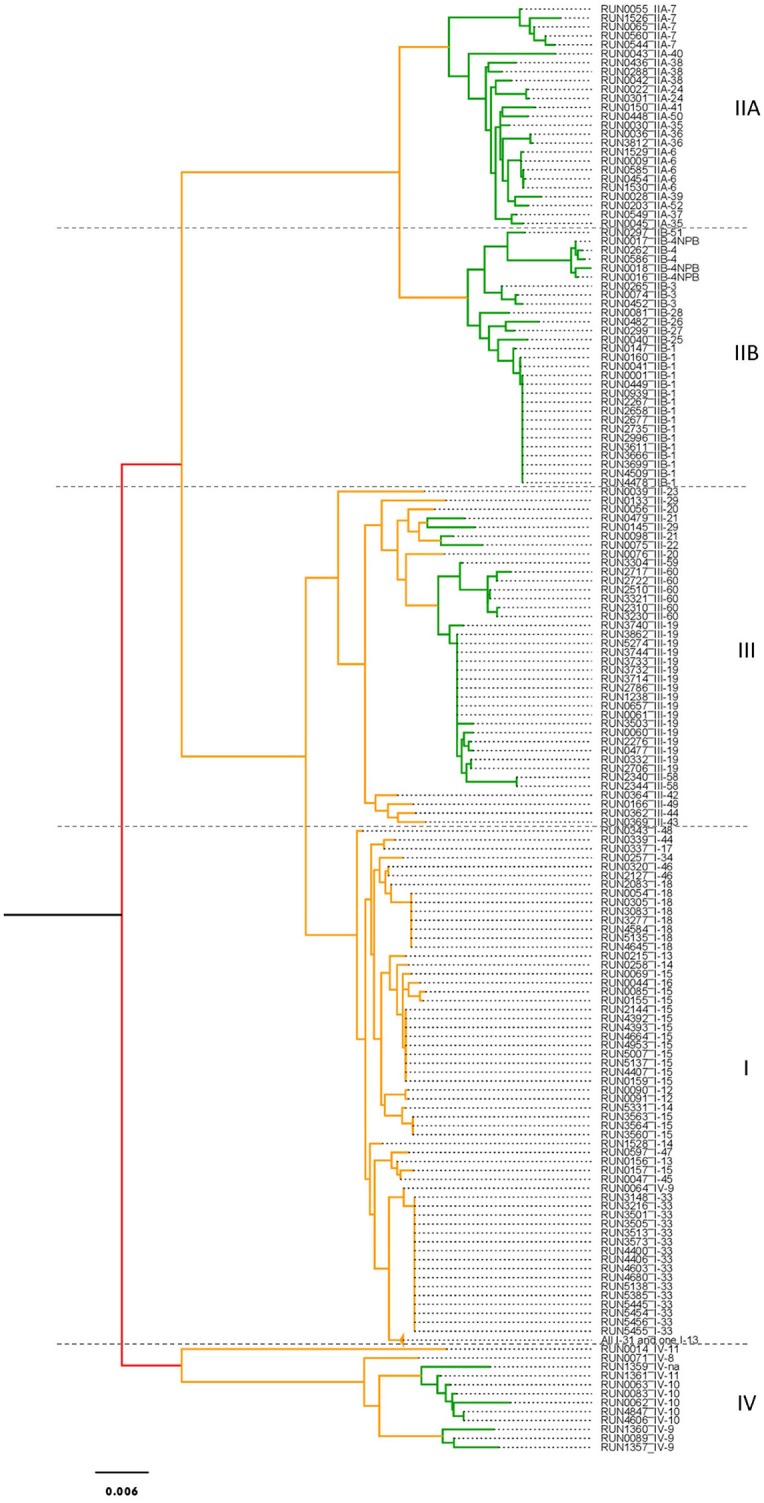
Phylogenetic tree based on the concatenated seven genes (*gdhA, gyrB, rplB, leuS, adk, mutS*, and *egl*). Supports of branch were generated by aLRT. Red branches show aLRT support value >0.5, orange between (0.2 ≤ × ≤ 0.5), green <0.2.

To reconstruct the phylogeny of the RSSC isolates, we first estimated using jModelTest2 for the best-fit substitution model for each gene and for concatenated sequences (with recombination regions removed; Table S5). We found that Tamura-Nei+G (for *gdhA, rplB* and *egl*), Tamura-Nei+I (for *leuS*), Hasegawa-Kishino-Yano+I (for *adk*), Hasegawa-Kishino-Yano+I+G (for *gyrB* and *mutS*) and TIM1+I+G (for concatenated sequences) were the best-fit substitution models (Table S5). Our phylogenetic analyses compared the Icong index values of the 8 phylogenetic trees (Figure 2). The Icong congruency index values indicated that all the trees were more congruent with each other than expected by chance (Table S6). The *rplB* and *leuS* trees were the most congruent (Icong = 7.441), and the concatenate and *adk* trees were the least congruent (Icong = 2.923). Overall, the concatenated tree was the least congruent with other genes (mean Icong = 3.284) than with isolates' genes (mean Icong: *gdhA* = 5.277, *gyrB* = 4.891, *rplB* = 5.612, *leuS* = 5.625, *adk* = 4.689, *mutS* = 5.391, *egl* = 4.195).

### Contrasting levels of RSSC genetic diversity in the SWIO islands

Based on the MLST data, genetic diversity was measured on worldwide (C2), SWIO (C1), and island (from C4 to C10) scales (Table 4). On the worldwide scale (C2, *n* = 90), a very high polymorphism was revealed for each of the 7 loci (H_E_ ranging from 0.94 to 0.98; mean H_E_ = 0.96; and mean A = 2.90). MLST showed very high discriminatory power (HGDI = 0.999), identifying 88 haplotypes, including 87 singletons that were epidemiologically unrelated between countries. On the SWIO scale (C1, *n* = 145), a high level of polymorphism was also revealed for each of the 7 loci, but logically to a lesser extent than on the worldwide scale (H_E_ ranging from 0.52 to 0.74; mean H_E_ = 0.64; and mean A = 2.11). The discriminatory power of MLST was high (HGDI = 0.94). The 145 isolates were resolved into 55 haplotypes with 30 singletons, suggesting the presence of some epidemiologically related strains between SWIO countries. On the island scale, contrasting levels of genetic diversity were observed. On the Comoros (Anjouan and Mohéli) (C7, *n* = 18) and Seychelles (Mahé and Praslin) (C8, *n* = 12) archipelagos, no genetic diversity was revealed. On the other islands, different levels of genetic diversity were shown, with high levels in Reunion, Mauritius, and Madagascar (C4, *n* = 51; C5, *n* = 29; and C6, *n* = 25, respectively; mean H_E_ ranging from 0.58 to 0.78; mean A ranging from 1.99 to 2.46), a moderate level in Mayotte (C9, *n* = 7; mean H_E_ = 0.45; mean A = 1.80) and a low level in Rodrigues (C10, *n* = 3; mean H_E_ = 0.31; mean A = 1.57; 3 loci were monomorphic). The discriminatory power of MLST varied depending on the SWIO islands (from HGDI = 0.65 for Reunion to HGDI = 0.96 for Mauritius).

**Table 4:** Measures of genetic variability of RSSC collections using MLST loci.

**Locus**	**C1**			**C2**			**C3**			**C4**			**C5**			**C6**			**C7**			**C8**			**C9**			**C10**		
	**Na**	**A**	**H_E_**	**Na**	**A**	**H_E_**	**Na**	**A**	**H_E_**	**Na**	**A**	**H_E_**	**Na**	**A**	**H_E_**	**Na**	**A**	**H_E_**	**Na**	**A**	**H_E_**	**Na**	**A**	**H_E_**	**Na**	**A**	**H_E_**	**Na**	**A**	**H_E_**
*gdhA*	8	1.87	0.52	36	2.84	0.94	40	2.37	0.67	4	1.91	0.55	3	1.49	0.31	6	2.52	0.81	1	1.00	0.00	1	1.00	0.00	2	1.43	0.26	1	1.00	0.00
*gyrB*	15	2.31	0.73	60	2.96	0.98	69	2.68	0.83	8	2.07	0.62	6	2.39	0.77	7	2.33	0.73	1	1.00	0.00	1	1.00	0.00	3	2.11	0.62	2	2.00	0.53
*rplB*	10	1.91	0.54	35	2.83	0.94	40	2.4	0.69	4	1.94	0.57	3	1.76	0.47	7	2.56	0.83	1	1.00	0.00	1	1.00	0.00	2	1.43	0.26	1	1.00	0.00
*leuS*	7	1.97	0.57	42	2.84	0.94	43	2.4	0.7	4	1.94	0.57	4	2.01	0.59	4	2.03	0.60	1	1.00	0.00	1	1.00	0.00	2	1.43	0.26	1	1.00	0.00
*adk*	15	2.32	0.73	53	2.93	0.97	61	2.67	0.83	6	2.01	0.60	5	2.35	0.75	11	2.74	0.89	1	1.00	0.00	1	1.00	0.00	3	2.11	0.62	2	2.00	0.53
*mutS*	10	2.07	0.62	48	2.91	0.96	52	2.52	0.75	5	1.99	0.59	4	1.98	0.59	7	2.40	0.76	1	1.00	0.00	1	1.00	0.00	3	1.86	0.48	2	2.00	0.53
*egl*	14	2.32	0.74	64	2.97	0.98	74	2.69	0.84	6	2.04	0.61	6	2.39	0.77	8	2.61	0.85	1	1.00	0.00	1	1.00	0.00	4	2.26	0.66	2	2.00	0.53
Number of isolates	145			90			235			51			29			25			18			12			7			3		
Pol. loci (%)	100			100			100			100			100			100			0			0			100			57.14		
Mean A	2.11			2.90			2.53			1.99			2.053			2.46			1.00			1.00			1.80			1.57		
Mean H_E_	0.64			0.96			0.76			0.58			0.605			0.78			0.00			0.00			0.45			0.31		
HGDI	0.94			1.00			0.98			0.65			0.967			0.96			0.00			0.00			0.86			0.67		
Haplotypes	55			88			145			10			17			19			1			1			5			2		
Singletons	30			87			124			6			10			15			0			0			4			1		

*Allele number (Na), allelic richness (A), Nei's unbiased diversity index (H_E_). Hunter-Gaston Diversity Index (HGDI): power of discrimination for the molecular typing methods. The SWIO collection (C1) is subdivided into seven sub-collections: 51 isolates from Reunion (C4), 29 from Mauritius, (C5), 25 from Madagascar (C6), 18 from Comoros (C7), 12 from Seychelles (C8), 7 from Mayotte (C9) and 3 from Rodrigues (C10). The worldwide collection (C2) includes RSSC strains from Africa (n = 23), America (n = 36), Asia (n = 28), Europe (n = 1), and Oceania (n = 2). The third collection, C3, corresponds to C1 + C2 (C3, n = 235)*.

### Genetic relationships between SWIO and worldwide RSSC strains

**Figure 3:**
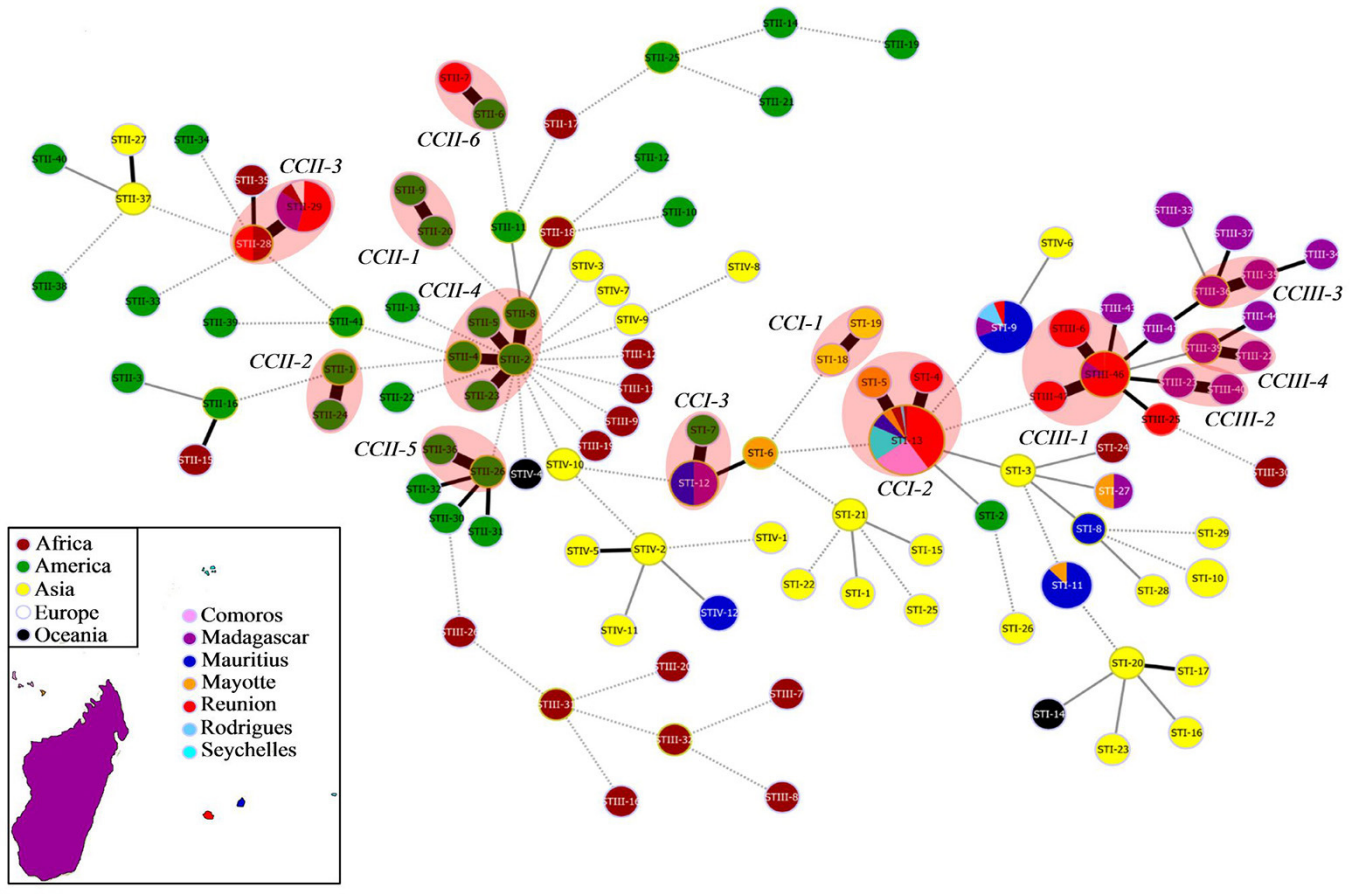
Minimum spanning tree (MST) from MLST data displaying genetic relationships between RSSC SWIO and worldwide reference strains (C3 collection, *n* = 235). MLST data are based on seven gene regions (*gdhA, mutS, adk, leuS, rplB, gyrB*, and *egl*). The dot colors indicate the country of isolation. Worldwide reference strains: Africa (brown), America (green), Asia (yellow), Oceania (black), Europe (white). SWIO strains: Reunion (red), Maurice (blue metallic), Comoros (pink), Madagascar (purple), Seychelles (turquoise), Rodrigues (sky blue) and Mayotte (orange). The labels in the dots indicate the MLST sequence type numbers (ST). Black thick lines, black regular lines, gray thin lines, and gray dashed lines joining haplotypes indicate single, double, triple, and more variations, respectively. No link is indicative of variations at >3 loci. Red halos denote a clonal complex (CC).

In the SWIO (C1, *n* = 145), MLST identified a total of 29 STs (Figure 3 and Table S1) that showed variable prevalence and geographic distribution, with some STs detected in several islands and other STs present in one island. The most striking observation was STI-13, which was detected in all SWIO islands except the Madagascar highlands and represented 48% of the isolates included in the SWIO RSSC collection. STI-13 was also the unique ST identified in the Comoros and Seychelles archipelagos. Some STs were represented by only one strain, such as STII-7 isolated in Reunion. Several STs were present only in one island such as STI-8 and STIV-12 in Mauritius, STI-4 and STII-7 in Reunion, STI-6 in Mayotte, and STIII-34 and STIII-37 in Madagascar. Moreover, MLST showed different levels of discrimination depending on the sequevars (from one to nine STs per sequevar). Only one ST was identified for sequevars I-13 (STI-4), I-14 (STI-8), I-15 (STI-11), I-33 (STI-9), I-46 (STI-27), IIA-36 (STII-7), III-58 (STIII-33) III-59 (STIII-34), and IV-10 (STIV-12). Sequevars I-18, I-31, and IIB-1 were subdivided into 2 STs (STI-6 and STI-12; STI-5 and STI-13; STII-28 and STII-29), respectively. Sequevars III-60 and III-19 were split into 5 STs (STIII-35, STIII-36, STIII-37, STIII-41, and STIII-44) and 9 STs (STIII-6, STIII-22, STIII-23, STIII-25, STIII-39, STIII-40, STIII-43, STIII-46, and STIII-47), respectively.

An MST was constructed to reveal the genetic relatedness between the SWIO and worldwide reference strains (C3, *n* = 235; Figure 3). A total of 110 STs were revealed, and 79 of these STs appeared as singletons. Thirteen CCs, linking STs by single-locus variants, were identified: 3 gathering phylotype I isolates (from CCI-1 to CCI-3), 6 grouping phylotype II isolates (from CCII-1 to CCII-6), and 4 comprising phylotype III isolates (from CCIII-1 to CCIII-4). No CCs with phylotype IV strains were identified. The number of STs per CC was variable, from 2 STs (such as CCI-1) to 5 STs (CCII-4). Interestingly, four of the 13 CCs appeared specific to the SWIO, all of these CCs including phylotype III isolates: CCIII-1 (sequevar III-19, Reunion and Madagascar), CCIII-2 (sequevar III-19, Madagascar), CCIII-3 (sequevars III-19 and III-60, Madagascar), and CCIII-4 (sequevar III-19, Madagascar). Four CCs were shared between the SWIO and other countries, highlighting the epidemiological links between the SWIO and Africa, Americas or Europe: CCI-2, containing the most prevalent sequevar I-31 strains (Comoros, Mauritius, Mayotte, Reunion, Rodrigues, Seychelles, Benin, Ivory Coast, Kenya, and South Africa), CCI-3 including sequevar I-18 strains (Mauritius, Madagascar, French Guiana), CCII-3 composed of sequevar IIB-1 strains (Reunion, Madagascar, Kenya, the Netherlands, and Nigeria) and CCII-6 containing sequevar IIA-36 strains (Reunion and Martinique). The remaining five CCs were not found in the SWIO: CCI-1 (China), CCII-1 (Brazil), CCII-2 (Trinidad), CCII-4 (Grenada, USA, Guatemala and Venezuela), and CCII-5 (Peru and Colombia).

## Discussion

To develop relevant BW control strategies in the SWIO area, knowledge of the genetic diversity of RSSC isolates and the dynamic structure of their populations is a prerequisite. Although the oldest RSSC isolates were collected in 1957 in Mauritius (RUN3501, 3505, 3510, and 3513, stored at the plant protection platform in Reunion), the genetic diversity of the RSSC isolates that are prevalent on the SWIO islands has been scarcely documented. In this study, we first conducted a broad sampling to cover crop production areas, mainly Solanaceae, in the SWIO islands (Comoros, Mauritius, Reunion, Rodrigues, and Seychelles). This sampling is the largest ever reported for BW epidemiosurveillance, yielding 1704 RSSC isolates from 129 plots. This large collection provided us with a solid foundation to apply MLSA/MLST to infer the phylogenetic relationships among RSSC strains, reveal the distribution and the level of RSSC genetic diversity, and conduct molecular epidemiological studies throughout the SWIO area. Interestingly, this approach confirmed the well-known epidemiological and genetic traits of the RSSC as well as revealing new ones.

### An unsuspected BW situation in the SWIO

Unexpectedly, this study revealed that the four phylotypes of the RSSC coexist in the SWIO, representing a unique situation for such a restricted geographical area in the world. We confirmed the occurrence of phylotypes I, IIB-1, and III (formerly known as race 1/biovar 3, race 3/biovar 2, and race 1/biovar 1, respectively; Girard et al., 1993; Dookun et al., 2001; Khoodoo et al., 2007, 2010; Wicker et al., 2012; unpublished data) and reported the presence of two strains of phylotype IV for the first time in the SWIO. Through an analysis of partial *egl* sequences (Fegan and Prior, 2005) and isolates from previous surveys (in Mayotte: Chesneau et al., submitted; and in Madagascar: Ravelomanantsoa, 2016; Ravelomanantsoa et al., submitted), we assigned the SWIO RSSC isolates to 14 sequevars. Phylotype I included 7 sequevars (I-13, I-14, I-15, I-18, I-31, I-33, and I-46), phylotype II included two sequevars (IIA-36 and IIB-1), phylotype III included four sequevars (III-19, III-58, III-59, and III-60), and phylotype IV included one sequevar (IV-10). The distribution and the frequencies of the phylotypes and the sequevars appeared very variable among the different SWIO islands.

In Reunion, we confirmed the BW situation, i.e., the geographic distribution and the frequencies of the phylotypes, reported from a previous survey conducted in the 1990s (Girard et al., 1993), although the sizes of the collections differed greatly between the two studies (780 vs. 118 isolates). Phylotype I, which was isolated from a wide host range throughout the islands in the lowlands and sometimes at higher altitudes up to 1,120 m, showed a higher prevalence (70%) than phylotypes II (22%) and III (8%). We assigned phylotype I isolates to 3 sequevars (I-13, I-31, and I-33), with a strikingly high prevalence of the I-31 strain (94%). Phylotypes II and III were isolated from a more limited host range and only in two small geographical areas: in the highlands in a region called “Plaine des Cafres” for sequevar IIB-1 isolates and at mid-altitudes in the western part of Reunion for phylotype III isolates. We assigned the phylotype III isolates as sequevar III-19, which was previously known in Reunion (Wicker et al., 2012). Rare phylotype II isolates were also found in the lowlands as reported previously (Girard et al., 1993). Interestingly, one of these isolates (RUN3512), assigned as sequevar IIA-36, appeared phylogenetically closely-related to a strain from Martinique (CFBP2957). However, compared to the previous survey (Girard et al., 1993), we did not find phylotype III isolates in the south of Reunion, which may be explained by the regression of the traditional cultivation of *Pelargonium* cv. *rosé* over the past several years in Reunion. On the other islands of the SWIO region, the occurrence of phylotypes IIB-1 and III was shown only in the Madagascar highlands (Ravelomanantsoa, 2016). However, phylotype III diversity in Madagascar was higher than in Reunion since 4 sequevars were found. Sequevar III-19 was shared with Reunion, but the remaining sequevars III-58, III-59, and III-60 were specific to Madagascar (Ravelomanantsoa, 2016; Ravelomanantsoa et al., submitted). The collection of these phylotype III isolates in the highlands or at mid-altitudes is a common ecological trait of these isolates, confirming a report in Cameroon (Mahbou Somo Toukam et al., 2009) although three isolates were disclosed in the Ivory Coast lowlands (N'guessan et al., 2012).

In Mauritius, we expected to collect phylotype IIB-1 isolates, since the last surveys reported potato brown rot outbreaks in several areas from 2005 to 2008 caused by race 3/biovar 2 strains that were probably introduced by latently infected potato tubers (Khoodoo et al., 2007, 2010). However, we revealed the occurrence of only phylotype I isolates among a wide host range (except for the two phylotype IV isolates, surprisingly), corresponding to the BW situation before these phylotype IIB-1 outbreaks in which only race 1/biovar 3 strains were described (Dookun et al., 2001). In this study, we report the broad phylogenetic diversity within phylotype I for the first time in Mauritius as we assigned 5 sequevars (I-14, I-15, I-18, I-31, and I-33), with some that were shared with other SWIO islands such as I-15 (with Mayotte), I-18 (with Mayotte and Madagascar), I-33 (with Reunion, Rodrigues and Madagascar), and I-31 (all SWIO islands, except the Madagascar highlands). In Mauritius, the absence of the detection of phylotype IIB-1 isolates despite our extensive survey (780 isolates from 35 plots throughout Mauritius) may be explained by an efficient strategy of elimination of infected potato tubers from the fields and the production chains coupled with the use of 7-year crop rotations with sugarcane (Khoodoo et al., 2007), a non-host plant for RSSC strains (Girard et al., 1993). Phylotype IIB-1 strains are known to be distributed worldwide by seed potato tubers (Janse, 1996) and occur in temperate regions and in tropical highlands because of their lower optimum temperature (Elphinstone, 2005), such as in the highlands of Reunion where they are well-adapted to hosts that are cropped in cold environments. Recently, the phylotype IIB-1 strain was demonstrated to be more competitive in tomato than two phylotype I strains under cool conditions and the results were reversed at warmer temperatures (Milling et al., 2009; Huerta et al., 2015). Furthermore, phylotype IIB-1 is thought to survive for less time than other phylotypes in the absence of potato due to their narrow host range (French, 1994). In the Mauritian tropical lowlands, these strains are certainly less adapted and less competitive than other RRSC strains such as those belonging to phylotype I and this may explain why we did not detect these strains during our survey. Surprisingly, in Mauritius, we also collected two phylotype IV isolates from the same potato plot, likely detected due to the large sampling fulfilled in this study. These isolates, assigned to sequevar IV-10 by *egl* gene sequencing, appeared phylogenetically close to Indonesian strains and distant from Japanese sequevar IV-8 strains, which are considered very highly virulent on potato, tobacco and groundnut (Suga et al., 2013). Recently, sequevar IV-8 isolates were detected on potatoes in India (Sagar et al., 2014). These phylotype IV isolates were revealed outside their areas of origin (Japan-Indonesia-Australia) for the first time and were probably introduced to Mauritius and India by latent infected potato tubers. Altogether, these studies highlight the importance of epidemiosurveillance networks for early detection to restrict the further dissemination of RSSC strains.

In the other SWIO islands, Comoros (Anjouan and Mohéli), Seychelles (Mahé and Praslin), and Rodrigues, where only scarce isolates were assigned as phylotype I before this study (unpublished data; Yahiaoui et al., 2016), we confirmed the occurrence of only phylotype I from 31 plots. In Rodrigues, sequevars I-31 and I-33 were found. In the Comoros and Seychelles archipelagos, despite the collection of RSSC isolates from 28 plots and from different plants (several species of Solanaceae and one Fabaceae), it was striking to observe that only sequevar I-31 was assigned. To our knowledge, this is the first time that a unique sequevar of the RSSC has been reported throughout a country (insular or continental).

In summary, this study revealed the high prevalence of phylotype I since this phylotype was isolated in all SWIO islands, representing 87% of the collected isolates. In Comoros, Seychelles, Rodrigues, and Mauritius (except for the two likely introduced phylotype IV isolates), this phylotype was identified exclusively, which has also been reported in Mayotte (Chesneau et al., submitted) and in another island in Asia and in Taiwan (Lin et al., 2014). The high prevalence of phylotype I in the SWIO area seems to be relevant since the most probable origin of this phylotype is predicted to be Eastern Africa or Asia (Wicker et al., 2012). Additionally, phylotype I has been reported to exhibit better fitness under tropical conditions (Buddenhagen, 1986; Elphinstone, 2005) and to affect the highest number of hosts among all phylotypes (Hayward, 1994).

### MLSA/MLST confirmed that phylotype I is the most recombinogenic lineage within the RSSC and highlighted widespread and specific SWIO RSSC lineages

We applied a MLSA/MLST scheme that has been particularly valuable for the inference of the genetic relatedness and the evolutionary history of the different RSSC phylotypes (I, IIA, IIB, III, and IV; Wicker et al., 2012), specifically the phylotype III (Ravelomanantsoa et al., 2016). In this study, we retained the seven genes (*gdhA, gyrB, rplB, leuS, adk, mutS*, and *egl*) chosen by Ravelomanantsoa et al. (2016) since *fliC* and *ppsA* were not amplified from any tested strains. Compared to the worldwide collection of strains (C2), the level of genetic diversity in the SWIO isolates (C1) was lower, but overall, a similar trend of RSSC genetic diversity was observed. According to the genetic diversity estimators (nucleotide diversity, the number of polymorphic sites, the number of haplotypes), the same genes presented the lowest and the highest diversities in both collections. Phylogenetic reconstructions on individual genes and on the concatenated sequences revealed incongruent topologies as it was already observed (Wicker et al., 2012), suggesting that recombination is a significant driving force for RSSC evolution. We confirmed that recombination is ubiquitous within the RSSC and that phylotype I is the most recombinogenic lineage (Wicker et al., 2012). Phylotype I has been considered to possess the highest evolutionary potential (Wicker et al., 2012) due to its high capacity for natural transformation and recombination (Coupat et al., 2008; Guidot et al., 2009), its large host range (Hayward, 1994), and its virulence plasticity as determined in tomato, eggplant, and pepper accessions (Lebeau et al., 2011). We detected 13 recombination events occurring across six of the seven genes studied, and three of them concerned SWIO isolates (phylotypes I and IV). The number of recombination events was lower than that was found (*n* = 21) previously on a worldwide RSSC collection (Wicker et al., 2012), but it could be explained by the fact that in this previous study six recombination events were detected in two genes, *fliC* and *ppsA*, not used in our study.

The discriminatory power of MLST appeared variable depending on the sequevars. Nine sequevars (I-13, I-14, I-15, I-33, I-46, IIA-36, III-58, III-59, and IV-10) appeared homogenous since MLST did not differentiate these sequevars into several STs. This result was also observed for phylogenetically closely related lineages such as the sequevar IIB-4 strains, including strains that are pathogenic on bananas (causing Moko disease) and strains that are not pathogenic on bananas (sequevar IIB-4NPB strains; Wicker et al., 2012). The more diverse III-60 and III-19 sequevars were split into 5 STs and 9 STs, respectively. This result is consistent with previous MLSA/MLST (Castillo and Greenberg, 2007; Wicker et al., 2012; Ravelomanantsoa et al., 2016) and comparative genomic hybridization (Guidot et al., 2007) studies that described phylotype III as highly diverse. The discriminatory power of MLST also appeared variable according to the SWIO islands, illustrating the contrasting levels of RSSC genetic diversity in the SWIO area. Although MLST showed high discrimination for isolates from Reunion, Madagascar, Maurice, Mayotte, and Rodrigues (HGDI > 0.65), it was striking to observe that no discrimination was obtained for the isolates from the Comoros and Seychelles archipelagos. A lack of discrimination by MLST was already reported in Cameroon, but with isolates from a single field (Ravelomanantsoa et al., 2016). In Comoros and Seychelles, the isolates were from 28 fields.

Our MSLT study highlighted widespread and specific SWIO RSSC lineages. MLST identified 110 STs on the worldwide scale, with most STs composed of isolates from only one country. Strikingly, only eight STs included isolates isolated from several countries, including the SWIO, demonstrating the epidemiological links between these countries. Among these eight lineages, STII-28 (strains from Nigeria and Reunion) and STII-29 (strains from the Netherlands, Madagascar, Kenya, and Reunion) formed a CC (CCII-3), which confirmed the clonal structure of sequevar IIB-1 (Cellier et al., 2012) and the long-distance migration of this lineage by contaminated potato tubers, for example, from Mediterranean countries to Western Europe (Janse, 1996) or by *Pelargonium zonale* cuttings, for instance, from Guatemala to the USA (Kim et al., 2002). The six remaining STs suggested the long-distance dissemination of phylotype I or phylotype III between the SWIO islands (STI-9, sequevar I-33; STI-11, sequevar 15; STI-12, sequevar I-18; STI-27, sequevar I-46; STIII-46, sequevar III-19) or between the SWIO islands and Africa (STI-13, sequevar I-31). Until now, no dissemination of phylotype I has been reported, although the long-distance migration of this lineage was suggested by an MLSA study based on a worldwide RSSC collection (Wicker et al., 2012). Interestingly, among the 110 STs identified on the worldwide scale, 26 STs were found only in the SWIO, highlighting the specificity of these lineages. Moreover, among the 13 CCs identified worldwide, four appeared to be geographically restricted to the SWIO. These four CCs included only phylotype III strains, three of them being specific to Madagascar (CCIII-2, CCIII-3, and CCIII-4). This result illustrated the epidemiological connections of some phylotype III strains between Reunion and Madagascar (CCIII-1) as already reported (Poussier et al., 2000a,b; Castillo and Greenberg, 2007; Wicker et al., 2012).

### One genetic lineage is widespread throughout the SWIO islands and is linked to african RSSC populations

Unexpectedly, we revealed the very high prevalence of one genetic lineage (sequevar I-31; STI-13) throughout the SWIO islands. This genetic lineage was distributed in all SWIO islands, except for the Madagascar highlands, and was detected in 48% of the sampled plots. However, its prevalence varied depending upon the islands. In the Comoros and Seychelles archipelagos, it was a unique genetic lineage, but in Reunion and Mayotte, it was the most prevalent genetic lineage (94 and 86%, respectively). In Mauritius, its prevalence was lower, representing only 19% of the tested collection. Whatever the level of prevalence, this overrepresentation obviously reflects a lineage that has strongly adapted to the SWIO environment. This adaptation is not recent since the oldest SWIO strain (RUN3510) of this lineage was isolated in 1957 in Mauritius.

This genetic lineage is not confined solely to SWIO as sequevar I-31 were also reported in Brazil (Rodrigues et al., 2012) and in some continental African countries, such as the Ivory Coast (N'guessan et al., 2012), Benin (Sikirou et al., 2015), the Democratic Republic of Congo, Uganda, South Africa (Carstensen et al., 2017), and Kenya (unpublished data). Our MLST study revealed that the sequevar I-31 isolates from Africa (Benin, the Ivory Coast, Kenya, and South Africa) and from the SWIO belonged to the same ST (STI-13), except for the recombinogenic isolate RUN1431 from Mayotte (STI-4), but they belonged to the same CC, highlighting the epidemiological links between these populations. In the future, since we do not have the Brazilian sequevar I-31 strains in our collection so far, it will be interesting to determine the genetic relationships between these South-American, African, and SWIO strains. This epidemiological link between continental Africa and the SWIO islands could have resulted from latently infected plant material transfers between Africa and the SWIO, but how it occurred remains an open question. Such dissemination has been widely documented for phylotype IIB by contaminated bananas, potatoes, and *Pelargonium* (Buddenhagen, 1986; Janse, 1996; Kim et al., 2002), but not for phylotype I, although the long-distance migration of this lineage has previously been suggested (Wicker et al., 2012).

Interestingly, as in the SWIO area, sequevar I-31 was also found to be the most widespread and prevalent sequevar in the Ivory Coast where a RSSC survey leading to a 168-strain collection was conducted (N'guessan et al., 2012). The higher prevalence of I-31 may be explained by better fitness in the environment than that of other RSSC strains. In addition to previous reports (N'guessan et al., 2012; Rodrigues et al., 2012; Carstensen et al., 2017), we showed that I-31 strains have the capacity to infect a wide range of hosts, including both herbaceous plants (Solanaceae, Geraniaceae, Begoniaceae, Fabaceae, Asteraceae) and woody plants such as Eucalyptus or *Casuarina equisetifolia*. Importantly, we also isolated this lineage on weeds such as *Solanum nigrum* and *Amaranthus viridis*, which could be very significant for the survival of this lineage during unfavorable seasons since this was already documented for phylotype IIB-1 strains (Hayward, 1994; Tusiime et al., 1998; Pradhanang et al., 2000). These weeds could then serve as latent carriers, enabling the I-31 lineage to persist in the environment. Moreover, in the Ivory Coast, three sequevar I-31 strains were characterized by a broad virulence spectrum and high aggressiveness levels on tomato and eggplant accessions and were assigned to the two highest virulent pathoprofiles e and f as defined by Lebeau et al. (2011) and N'guessan et al. (2012). Only the emerging sequevar IIB4-NPB strains, detected first in Martinique and having a wide host range (Wicker et al., 2007), were as virulent as sequevar I-31 strains from the Ivory Coast (N'guessan et al., 2012). In Mayotte, when tested on tomatoes, sequevar I-31 strains were clustered into the most aggressive pathotypes, T-2 and T-3, referring to the classification of Lebeau et al. (2011), and the strains of sequevars I-18, I-46, and I-15 were clustered into the least aggressive pathotype, T-1 (Chesneau et al., submitted). Pathogenicity tests will have to be performed to verify whether the SWIO sequevar I-31 strains have a broader virulence spectrum and higher aggressiveness than the other genetic lineages identified throughout the SWIO area. Another interesting point is the fact that sequevar I-31 seems to be adapted to both warm and cool temperatures. Indeed, this lineage presents the largest range of altitude of isolation in our study because these strains were isolated in the lowlands and also in the highlands up to 1,120 m, confirming the findings in the Ivory Coast where these strains were isolated from the lowlands and up to altitudes above 1,000 m (N'guessan et al., 2012). In the SWIO, this feature is unique since all other sequevars were confined to the lowlands (< 500 m; phylotype I and one phylotype IIA strain), mid-altitudes (from 500 to 1,000 m; phylotype III) or the highlands (>1,200 m; phylotype IIB-1). Another potential explanation for this wide distribution is that this lineage produced different bacteriocins, similar to other RSSC strains (Arwiyanto et al., 1993), allowing this lineage to outcompete other RSSC strains in the environment as demonstrated by phylotype I strains that specifically inhibited phylotype IIB-1 strains in tomato rhizospheres and stems (Huerta et al., 2015). Further *in vitro* and *in planta* studies need to be conducted to verify whether the sequevar I-31 lineage produces bacteriocins that allow it to outcompete other SWIO genetic lineages. Such antagonism involving bacteriocin-like compounds has already been observed in fields where *Xanthomonas performans* became predominant relative to *Xanthomonas euvesicatoria* (Hert et al., 2005).

## Conclusion

This was the first large-scale study of RSSC in the SWIO area. MLSA/MLST has greatly expanded our knowledge regarding the phylogenetic and the genetic diversity of the RSSC in this area. We confirmed the occurrence of phylotypes I, II, and III and highlighted their variable distribution and frequencies on the SWIO islands and reported the presence of phylotype IV isolates that were likely introduced by infected potato tubers in Mauritius for the first time. Among all genetic lineages identified in the SWIO, we revealed the unexpectedly high prevalence of sequevar I-31 (STI-13), which occurred in all SWIO islands (except the Madagascar highlands) and exhibited genetic links with strains from continental Africa. Further studies are now required to understand the ecology of this lineage and why it is so strongly adapted to the SWIO environment. Further population genetic studies on different scales are also necessary to reconstruct the evolutionary history of this lineage and to decipher the epidemiological relationships between the SWIO and African populations. Such studies could be conducted with more conclusive methods such as the multilocus variable number tandem repeat analysis (MLVA), which was developed to study RSSC populations from cropping areas to continents (N'guessan et al., 2013; Parkinson et al., 2013; Ravelomanantsoa et al., 2016; Guinard et al., 2017). All of the MLST sequences generated by this study will be deposited soon in an open-access database dedicated to RSSC useful for BW epidemiosurveillance. Finally, this study is a starting point for the epidemiological surveillance of RSSC and will contribute to BW management in the SWIO.

## Author contributions

NY: conceived and designed the experiments, performed the experiments, analyzed the data, wrote the paper, and prepared the figures and tables; J-JC: conceived and designed the experiments; SR: provided the unpublished Madagascar strains; AH, BP, RJ, YJ-F, JéF, and JaF: participated in the surveys; BH, FG, GC, PP, and SP: conceived and designed the experiments and revised the manuscript.

### Conflict of interest statement

The authors declare that the research was conducted in the absence of any commercial or financial relationships that could be construed as a potential conflict of interest.

## Supplementary material

Figure S1 Variation in number of records for RSSC hosts in the SWIO area. Letters above bars represent groups of hosts (a–d) calculated by Chi-Square test by R software. Phylotype I (blue), phylotype II (orange), phylotype III (red), and phylotype IV (gray).

Figure S2 Split graphs of multilocus sequence analysis of the global collection (C3) for the seven loci (*gdhA, gyrB, rplB, leuS, adk, mutS*, and *egl*) and the data set of the concatenated sequences. Strains from each phylotype (I, IIA, IIB, III, and IV) of the RSSC are highlighted in blue, light green, dark green, red and gray, respectively.

Table S1 RSSC strains used in this study and corresponding phylogenetic and genotyping data.

Table S2 Description of MLST loci, corresponding oligonucleotide primers, and PCR conditions.

Table S3 Genetic properties of the loci used in MLSA for each phylotype of the SWIO (C1) and the worldwide (C2) RSSC collections.

Table S4 GenBank accession numbers for the partial sequences of seven loci used in this study. New accession numbers of sequences are in bold.

Table S5 Substitution models of the seven genes studied using Jmodeltest 2.

Table S6 Comparison between the seven genes (*gdhA, gyrB, rplB, leuS, adk, mutS*, and *egl*) and the concatenated-genes trees generated by the congruency Index Icong values. Icong, *p*-value, and MAST were indicated in this table.
