# Supplementary figures and images for: Genetic Diversity of the Ralstonia solanacearum Species Complex in the Southwest Indian Ocean Islands
Source: Front Plant Sci. 2017 Dec 19;8:2139. doi: 10.3389/fpls.2017.02139 (PMC5742265; doi:10.3389/fpls.2017.02139)

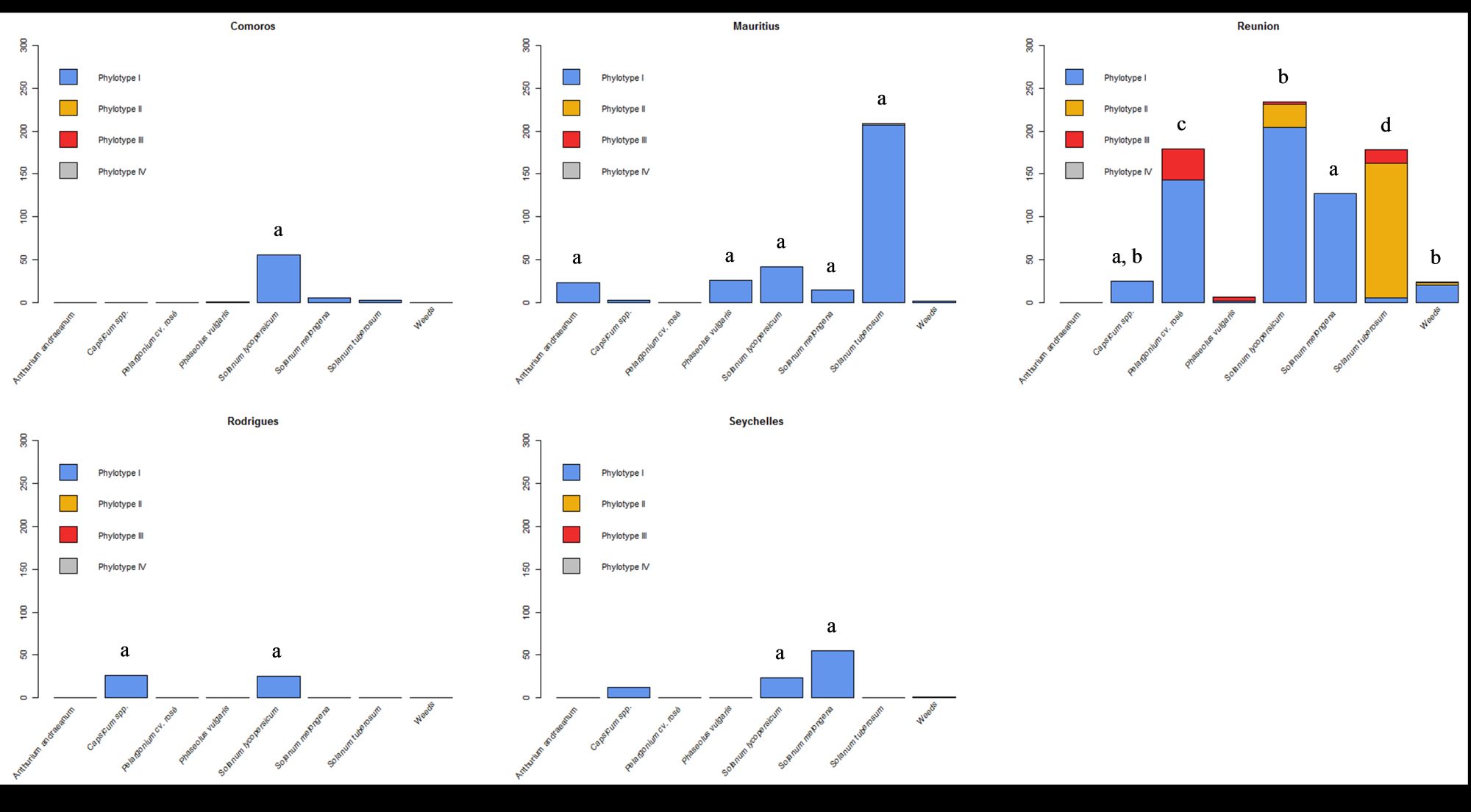

Supplement: Figure S1 — Variation in number of records for RSSC hosts in the SWIO area. Letters above bars represent groups of hosts (a–d) calculated by Chi-Square test by R software. Phylotype I (blue), phylotype II (orange), phylotype III (red), and phylotype IV (gray). [file Image1.JPEG]

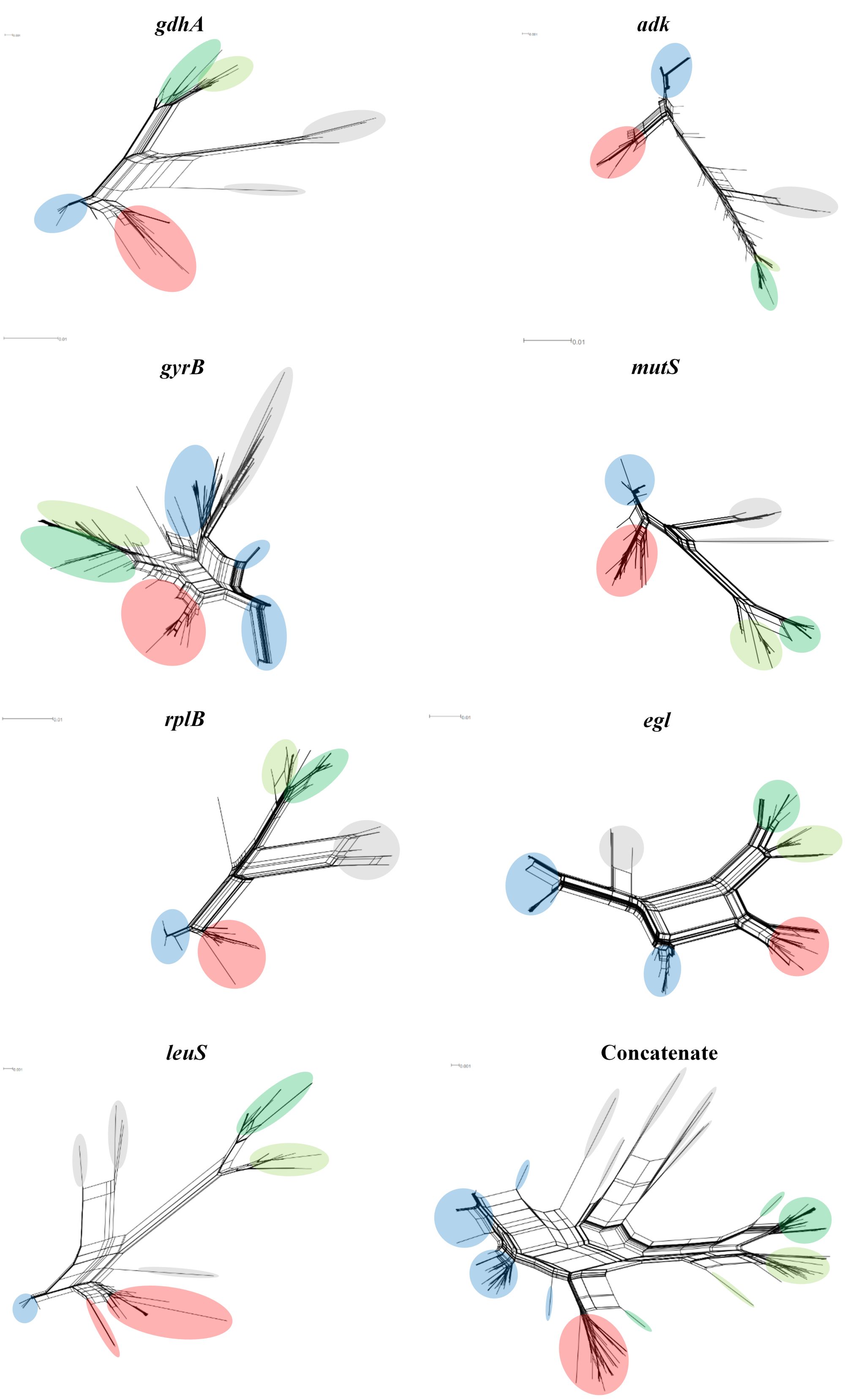

Supplement: Figure S2 — Split graphs of multilocus sequence analysis of the global collection (C3) for the seven loci (gdhA, gyrB, rplB, leuS, adk, mutS, and egl) and the data set of the concatenated sequences. Strains from each phylotype (I, IIA, IIB, III, and IV) of the RSSC are highlighted in blue, light green, dark green, red and gray, respectively. [file Image2.JPEG]
