# Supplementary material for: Genetic Diversity of the Ralstonia solanacearum Species Complex in the Southwest Indian Ocean Islands
Source: Front Plant Sci. 2017 Dec 19;8:2139. doi: 10.3389/fpls.2017.02139 (PMC5742265; doi:10.3389/fpls.2017.02139)
Supplement: Table S1 — RSSC strains used in this study and corresponding phylogenetic and genotyping data. [file Table1.DOCX]

| **Collection** | **RUN ID** | **Lab ID** | **Phylotype** | **Sequevar** | **ST** | **Geographic origin** | **Year** | **Host of isolation** | ***gdhA*** | ***gyrB*** | ***rplB*** | ***leuS*** | ***adk*** | ***mutS*** | ***egl*** |
| --- | --- | --- | --- | --- | --- | --- | --- | --- | --- | --- | --- | --- | --- | --- | --- |
| C2 | RUN0001 | IPO1609 | IIB | 1 | STII-29 | Netherlands | 1995 | *Solanum tuberosum* | 21 | 26 | 23 | 19 | 19 | 30 | 50 |
| C2 | RUN0009 | A3909 | IIA | 6 | STII-8 | Usa | 1989 | *Heliconia rostrata* | 45 | 76 | 43 | 48 | 63 | 23 | 21 |
| C2 | RUN0014 | ACH732 | IV | 11 | STIV-4 | Australia | 1978 | *Solanum lycopersicum* | 46 | 61 | 34 | 40 | 64 | 31 | 32 |
| C2 | RUN0016 | CFBP6784 | IIB | 4NPB | STII-30 | Martinique | 1999 | *Anthurium andreanum* | 37 | 15 | 38 | 45 | 65 | 57 | 68 |
| C2 | RUN0017 | CFBP6783 | IIB | 4NPB | STII-31 | Martinique | 2002 | *Heliconia caribea* | 37 | 28 | 38 | 45 | 66 | 57 | 68 |
| C2 | RUN0018 | ANT80 | IIB | 4NPB | STII-32 | Martinique | 2002 | *Anthurium andreanum* | 37 | 29 | 38 | 45 | 58 | 57 | 68 |
| C2 | RUN0022 | B34 | IIA | 24 | STII-9 | Brazil | 1998 | *Musa sp.* | 38 | 70 | 39 | 46 | 67 | 58 | 70 |
| C2 | RUN0028 | CFBP2958 | IIA | 39 | STII-10 | Guadeloupe | 1985 | *Solanum lycopersicum* | 36 | 30 | 35 | 24 | 63 | 53 | 39 |
| C2 | RUN0030 | CFBP2972 | IIA | 35 | STII-11 | Martinique | 1986 | *Solanum tuberosum* | 39 | 31 | 43 | 48 | 63 | 23 | 40 |
| C2 | RUN0036 | CFBP2957 | IIA | 36 | STII-6 | Martinique | 1987 | *Solanum lycopersicum* | 39 | 17 | 19 | 17 | 16 | 23 | 49 |
| C2 | RUN0039 | CFBP3059 | III | 23 | STIII-9 | Burkina Faso | 1990 | *Solanum melongena* | 1 | 2 | 2 | 13 | 13 | 17 | 1 |
| C2 | RUN0040 | CIP10 | IIB | 25 | STII-33 | Peru | 1979 | *Solanum tuberosum* | 21 | 32 | 23 | 25 | 27 | 32 | 35 |
| C2 | RUN0041 | CIP117 | IIB | 1 | STII-28 | Nigeria | 1968 | *Solanum tuberosum* | 21 | 72 | 23 | 19 | 19 | 30 | 50 |
| C2 | RUN0042 | CIP120 | IIA | 38 | STII-12 | Peru | 1979 | *Solanum tuberosum* | 36 | 68 | 43 | 26 | 28 | 58 | 62 |
| C2 | RUN0043 | CIP239 | IIA | 40 | STII-13 | Brazil | 1983 | *Solanum tuberosum* | 47 | 33 | 27 | 48 | 29 | 33 | 37 |
| C2 | RUN0044 | ACH92 | I | 16 | STI-14 | Australia | 1966 | *Zingiber officinale* | 23 | 71 | 20 | 20 | 30 | 34 | 23 |
| C2 | RUN0045 | CIP301 | IIA | 35 | STII-14 | Peru | 1988 | *Solanum tuberosum* | 43 | 34 | 43 | 46 | 31 | 59 | 55 |
| C2 | RUN0047 | CIP365 | I | 45 | STI-15 | Philippines | 1989 | *Solanum tuberosum* | 19 | 66 | 40 | 22 | 17 | 35 | 26 |
| C2 | RUN0054 | GMI1000 | I | 18 | STI-7 | French Guiana | 1978 | *Solanum lycopersicum* | 22 | 25 | 22 | 21 | 17 | 29 | 52 |
| C2 | RUN0055 | ICMP7963 | IIA | 7 | STII-15 | Kenya | 1960 | *Solanum tuberosum* | 40 | 75 | 37 | 43 | 57 | 54 | 36 |
| C2 | RUN0056 | J25 | III | 20 | STIII-16 | Kenya | 1998 | *Solanum tuberosum* | 14 | 3 | 15 | 2 | 11 | 8 | 12 |
| C4 | RUN0060 | JT525 | III | 19 | STIII-25 | Reunion | 1993 | *Pelargonium cv. rosé* | 3 | 7 | 13 | 11 | 14 | 7 | 11 |
| C4 | RUN0061 | JT528 | III | 19 | STIII-6 | Reunion | 1993 | *Solanum tuberosum* | 3 | 5 | 13 | 11 | 4 | 7 | 11 |
| C2 | RUN0062 | R229 | IV | 10 | STIV-1 | Indonesia | 1988 | *Banana sp.* | 31 | 58 | 33 | 39 | 52 | 28 | 28 |
| C2 | RUN0063 | R230 | IV | 10 | STIV-5 | Indonesia | 1998 | *Musa sp.* | 20 | 35 | 21 | 18 | 32 | 28 | 28 |
| C2 | RUN0064 | JT663 | IV | 9 | STIV-6 | Indonesia | 1998 | *Syzygium aromaticum* | 23 | 36 | 22 | 22 | 62 | 26 | 65 |
| C2 | RUN0065 | CFBP2047 | IIA | 7 | STII-16 | Usa | 1953 | *Solanum lycopersicum* | 40 | 67 | 37 | 43 | 57 | 54 | 54 |
| C2 | RUN0069 | MAFF211266 | I | 15 | STI-16 | Japan | nd | *Solanum lycopersicum* | 23 | 71 | 20 | 20 | 33 | 36 | 79 |
| C2 | RUN0071 | MAAF301558 | IV | 8 | STIV-7 | Japan | nd | *Solanum tuberosum* | 26 | 37 | 28 | 27 | 34 | 37 | 31 |
| C2 | RUN0074 | MOLK2 | IIB | 3 | STII-27 | Philippines | 1991 | *Musa sp.* | 21 | 59 | 41 | 47 | 53 | 62 | 69 |
| C2 | RUN0075 | NCPPB0332 | III | 22 | STIII-26 | Zimbabwe | 1954 | *Solanum tuberosum* | 4 | 15 | 3 | 9 | 11 | 13 | 13 |
| C2 | RUN0076 | NCPPB0342 | III | 20 | STIII-30 | Zimbabwe | nd | *Nicotinia tabacum* | 6 | 8 | 1 | 16 | 14 | 3 | 9 |
| C2 | RUN0081 | NCPPB3987 | IIB | 28 | STII-34 | Brazil | nd | *Solanum tuberosum* | 21 | 68 | 21 | 28 | 35 | 38 | 34 |
| C2 | RUN0083 | PSI07 | IV | 10 | STIV-2 | Indonesia | nd | *Solanum lycopersicum* | 20 | 60 | 21 | 18 | 54 | 28 | 28 |
| C2 | RUN0085 | PSS190 | I | 15 | STI-17 | Taiwan | 1995 | *Solanum lycopersicum* | 23 | 71 | 20 | 20 | 36 | 55 | 80 |
| C2 | RUN0089 | R28 | IV | 9 | STIV-8 | Indonesia | nd | *Syzygium aromaticum* | 42 | 38 | 42 | 44 | 37 | 39 | 75 |
| C2 | RUN0090 | R288 | I | 12 | STI-18 | China | nd | *Morus alba* | 41 | 69 | 22 | 22 | 17 | 61 | 22 |
| C2 | RUN0091 | R292 | I | 12 | STI-19 | China | nd | *Morus alba* | 41 | 69 | 22 | 22 | 38 | 61 | 22 |
| C2 | RUN0098 | K179 | III | 21 | STIII-20 | Zimbabwe | 1959 | *Nicotinia tabacum* | 17 | 11 | 4 | 12 | 11 | 15 | 15 |
| C2 | RUN0100 | UW308 | I | 31 | STI-13 | Kenya | 1974 | *Capsicum annuum* | 23 | 20 | 22 | 22 | 20 | 29 | 43 |
| C2 | RUN0104 | UW395 | I | 31 | STI-13 | South Africa | 1986 | *Solanum lycopersicum* | 23 | 20 | 22 | 22 | 20 | 29 | 43 |
| C2 | RUN0133 | CMR15 | III | 29 | STIII-7 | Cameroon | 2005 | *Solanum lycopersicum* | 9 | 14 | 5 | 1 | 10 | 1 | 18 |
| C2 | RUN0145 | CFBP6942 | III | 29 | STIII-12 | Cameroon | 2005 | *Solanum scabrum* | 15 | 1 | 16 | 4 | 15 | 10 | 20 |
| C2 | RUN0147 | CFBP7029 | IIB | 1 | STII-35 | Cameroon | 2005 | *Solanum lycopersicum* | 21 | 72 | 23 | 19 | 39 | 40 | 50 |
| C2 | RUN0150 | CFBP7032 | IIA | 41 | STII-17 | Cameroon | 2005 | *Solanum lycopersicum* | 39 | 75 | 43 | 29 | 63 | 41 | 58 |
| C2 | RUN0155 | PSS366 | I | 15 | STI-20 | Taiwan | 2003 | *Solanum lycopersicum* | 23 | 71 | 20 | 20 | 40 | 55 | 46 |
| C2 | RUN0156 | PSS216 | I | 13 | STI-21 | Taiwan | 1996 | *Solanum lycopersicum* | 19 | 39 | 40 | 22 | 17 | 29 | 29 |
| C2 | RUN0157 | PSS4 | I | 15 | STI-22 | Taiwan | 1988 | *Solanum lycopersicum* | 19 | 40 | 40 | 30 | 17 | 42 | 46 |
| C2 | RUN0159 | PSS358 | I | 15 | STI-23 | Taiwan | 2003 | *Solanum lycopersicum* | 27 | 66 | 20 | 20 | 22 | 55 | 46 |
| C4 | RUN0160 | JT516 | IIB | 1 | STII-28 | Reunion | 1993 | *Solanum tuberosum* | 21 | 72 | 23 | 19 | 19 | 30 | 50 |
| C2 | RUN0166 | CFBP7038 | III | 49 | STIII-8 | Cameroon | 2005 | *Solanum scabrum* | 10 | 9 | 7 | 15 | 10 | 2 | 5 |
| C2 | RUN0203 | CFBP7054 | IIA | 52 | STII-18 | Cameroon | 2005 | *Solanum lycopersicum* | 36 | 41 | 43 | 48 | 63 | 23 | 61 |
| C2 | RUN0215 | CFBP7058 | I | 13 | STI-24 | Cameroon | 2005 | *Solanum scabrum* | 23 | 42 | 22 | 22 | 22 | 43 | 78 |
| C2 | RUN0257 | PSS219 | I | 34 | STI-25 | Taiwan | 1996 | *Solanum lycopersicum* | 19 | 43 | 40 | 22 | 22 | 55 | 45 |
| C2 | RUN0258 | PSS81 | I | 14 | STI-26 | Taiwan | nd | *Solanum lycopersicum* | 28 | 71 | 29 | 22 | 41 | 29 | 71 |
| C2 | RUN0262 | UW170 | IIB | 4 | STII-36 | Colombia | 1961 | *Heliconia rostrata* | 37 | 74 | 38 | 45 | 42 | 57 | 68 |
| C2 | RUN0265 | CIP418 | IIB | 3 | STII-37 | Indonesia | 1991 | *Arachis hypogaea* | 21 | 73 | 41 | 47 | 43 | 62 | 69 |
| C2 | RUN0288 | CFBP6779 | IIA | 38 | STII-19 | Martinique | 2002 | *Canna indica* | 29 | 44 | 43 | 46 | 44 | 44 | 59 |
| C2 | RUN0297 | CFBP7014 | IIB | 51 | STII-38 | Trinidad | 2006 | *Anthurium andreanum* | 21 | 73 | 30 | 31 | 45 | 45 | 33 |
| C2 | RUN0299 | IBSBF1712 | IIB | 27 | STII-39 | Brazil | 2002 | *Pelargonium cv. rosé* | 21 | 45 | 23 | 32 | 46 | 56 | 57 |
| C2 | RUN0301 | IBSBF1900 | IIA | 24 | STII-20 | Brazil | 2000 | *Musa sp.* | 38 | 70 | 39 | 46 | 63 | 58 | 70 |
| C6 | RUN0305 | MAD-002 | I | 18 | STI-12 | Madagascar | 2006 | *Solanum lycopersicum* | 22 | 25 | 22 | 21 | 17 | 29 | 42 |
| C6 | RUN0320 | MAD-017 | I | 46 | STI-27 | Madagascar | 2006 | *Capsicum annuum* | 23 | 23 | 22 | 22 | 22 | 24 | 48 |
| C6 | RUN0332 | MAD-029 | III | 19 | STIII-23 | Madagascar | 2006 | *Solanum tuberosum* | 3 | 22 | 12 | 11 | 1 | 7 | 11 |
| C2 | RUN0337 | P11 | I | 17 | STI-28 | China | nd | *Arachis hypogaea* | 23 | 46 | 22 | 22 | 47 | 25 | 27 |
| C2 | RUN0339 | O3 | I | 44 | STI-29 | China | nd | *Olea europae* | 23 | 47 | 22 | 33 | 48 | 25 | 63 |
| C2 | RUN0343 | M02 | I | 48 | STI-3 | China | nd | *Morus alba* | 23 | 48 | 22 | 22 | 22 | 29 | 24 |
| C2 | RUN0362 | DGBBC1138 | III | 44 | STIII-31 | Guinea | nd | *Solanum tuberosum* | 7 | 9 | 18 | 7 | 11 | 21 | 3 |
| C2 | RUN0364 | DGBBC1227 | III | 42 | STIII-11 | Guinea | nd | *Solanum tuberosum* | 13 | 10 | 10 | 6 | 12 | 19 | 8 |
| C2 | RUN0369 | DGBBC1125 | III | 43 | STIII-32 | Guinea | nd | *Solanum tuberosum* | 8 | 9 | 17 | 8 | 10 | 18 | 4 |
| C2 | RUN0436 | 6035 | IIA | 38 | STII-21 | French Guiana | 2006 | Water | 43 | 75 | 31 | 34 | 49 | 53 | 60 |
| C2 | RUN0448 | T1-UY | IIA | 50 | STII-22 | Uruguay | 2004 | *Solanum lycopersicum* | 30 | 49 | 43 | 48 | 16 | 46 | 38 |
| C2 | RUN0449 | UW551 | IIB | 1 | STII-29 | Kenya | 2003 | *Pelargonium cv. rosé* | 21 | 26 | 23 | 19 | 19 | 30 | 50 |
| C2 | RUN0452 | CFBP1409 | IIB | 3 | STII-40 | Honduras | 1965 | *Musa sp.* | 21 | 50 | 41 | 47 | 50 | 62 | 81 |
| C2 | RUN0454 | UW181 | IIA | 6 | STII-23 | Venezuela | 1960 | *Banana plantain* | 45 | 76 | 43 | 35 | 61 | 23 | 21 |
| C4 | RUN0471 | JT519 | I | 31 | STI-13 | Reunion | 1993 | *Pelargonium cv. rosé* | 23 | 20 | 22 | 22 | 20 | 29 | 43 |
| C6 | RUN0477 | CFBP734 | III | 19 | STIII-22 | Madagascar | 1997 | *Solanum tuberosum* | 3 | 13 | 11 | 11 | 6 | 7 | 11 |
| C2 | RUN0479 | NCPPB1018 | III | 21 | STIII-19 | Angola | 1961 | *Solanum tuberosum* | 16 | 12 | 6 | 3 | 7 | 14 | 16 |
| C2 | RUN0482 | CIP240 | IIB | 26 | STII-41 | Brazil | 1983 | *Solanum tuberosum* | 21 | 70 | 23 | 36 | 57 | 56 | 56 |
| C8 | RUN0487 | LD74 | I | 31 | STI-13 | Seychelles | 2007 | *Solanum melongena* | 23 | 20 | 22 | 22 | 20 | 29 | 43 |
| C8 | RUN0504 | LD091 | I | 31 | STI-13 | Seychelles | 2007 | *Capsicum annuum* | 23 | 20 | 22 | 22 | 20 | 29 | 43 |
| C2 | RUN0544 | RF30 | IIA | 7 | STII-24 | Trinidad | 2003 | *Solanum lycopersicum* | 44 | 67 | 37 | 49 | 60 | 60 | 67 |
| C2 | RUN0549 | RF38 | IIA | 37 | STII-25 | Trinidad | 2003 | *Solanum lycopersicum* | 43 | 75 | 43 | 17 | 60 | 59 | 41 |
| C2 | RUN0560 | RF66 | IIA | 7 | STII-1 | Trinidad | 2003 | *Solanum lycopersicum* | 44 | 67 | 37 | 49 | 57 | 60 | 67 |
| C2 | RUN0585 | GMI8044 | IIA | 6 | STII-2 | Grenada | 1984 | *Musa sp.* | 45 | 76 | 43 | 48 | 61 | 23 | 21 |
| C2 | RUN0586 | UW163 | IIB | 4 | STII-26 | Peru | 1967 | *Banana plantain* | 37 | 74 | 38 | 45 | 51 | 57 | 68 |
| C2 | RUN0597 | GMI8254 | I | 47 | STI-1 | Indonesia | nd | *Solanum lycopersicum* | 19 | 52 | 40 | 37 | 17 | 29 | 25 |
| C4 | RUN0608 | JT523 | I | 13 | STI-4 | Reunion | nd | *Solanum tuberosum* | 23 | 20 | 22 | 22 | 20 | 29 | 77 |
| C4 | RUN0657 | CFBP2146 | III | 19 | STIII-6 | Reunion | 1980 | *Pelargonium cv. rosé* | 3 | 5 | 13 | 11 | 4 | 7 | 11 |
| C4 | RUN0939 | JQ1118 | IIB | 1 | STII-29 | Reunion | 1988 | *Solanum tuberosum* | 21 | 26 | 23 | 19 | 19 | 30 | 50 |
| C4 | RUN1238 | CFBP4963 | III | 19 | STIII-6 | Reunion | 1999 | *Solanum tuberosum* | 3 | 5 | 13 | 11 | 4 | 7 | 11 |
| C2 | RUN1357 | UW519 | IV | 9 | STIV-9 | Indonesia | 1978 | *Syzygium aromaticum* | 42 | 53 | 32 | 44 | 61 | 63 | 75 |
| C2 | RUN1359 | UW521 | IV | na | STIV-10 | Indonesia | 1987 | *Syzygium aromaticum* | 20 | 54 | 21 | 21 | 61 | 29 | 64 |
| C2 | RUN1360 | R001 | IV | 9 | STIV-3 | Indonesia | 1990 | *Syzygium aromaticum* | 35 | 64 | 36 | 42 | 56 | 52 | 66 |
| C2 | RUN1361 | UW522 | IV | 11 | STIV-11 | Indonesia | 1985 | *Syzygium aromaticum* | 20 | 55 | 21 | 18 | 59 | 28 | 30 |
| C9 | RUN1431 | 07.040.3 | I | 31 | STI-5 | Mayotte | 2006 | *Solanum lycopersicum* | 23 | 20 | 22 | 22 | 20 | 30 | 43 |
| C9 | RUN1432 | 07.040.6 | I | 31 | STI-13 | Mayotte | 2006 | *Solanum lycopersicum* | 23 | 20 | 22 | 22 | 20 | 29 | 43 |
| C9 | RUN1439 | 07.040.21 | I | 31 | STI-13 | Mayotte | 2006 | *Solanum lycopersicum* | 23 | 20 | 22 | 22 | 20 | 29 | 43 |
| C2 | RUN1526 | AW1 | IIA | 7 | STII-3 | Usa | 1987 | *Solanum lycopersicum* | 40 | 56 | 37 | 43 | 57 | 47 | 53 |
| C2 | RUN1528 | UW568 | I | 14 | STI-2 | Guatemala | 2004 | *Solanum lycopersicum* | 23 | 57 | 22 | 22 | 57 | 29 | 71 |
| C2 | RUN1529 | UW588 | IIA | 6 | STII-4 | Guatemala | 2004 | *Musa sp.* | 45 | 76 | 43 | 48 | 57 | 23 | 21 |
| C2 | RUN1530 | UW595 | IIA | 6 | STII-5 | Guatemala | 2004 | *Musa sp.* | 45 | 76 | 43 | 48 | 18 | 23 | 21 |
| C2 | RUN1740 | CIV19 | I | 31 | STI-13 | Ivory Coast | 2010 | *Solanum melongena* | 23 | 20 | 22 | 22 | 20 | 29 | 43 |
| C9 | RUN1930 | ech7 | I | 31 | STI-13 | Mayotte | 2011 | *Solanum lycopersicum* | 23 | 20 | 22 | 22 | 20 | 29 | 43 |
| C9 | RUN2083 | YT11.107 | I | 18 | STI-6 | Mayotte | 2012 | *Capsicum frutescens* | 22 | 23 | 22 | 22 | 17 | 29 | 42 |
| C9 | RUN2127 | YT16.163 | I | 46 | STI-27 | Mayotte | 2012 | *Capsicum annuum* | 23 | 23 | 22 | 22 | 22 | 24 | 48 |
| C9 | RUN2144 | YT19.185 | I | 15 | STI-11 | Mayotte | 2012 | *Solanum melongena* | 23 | 19 | 20 | 20 | 22 | 29 | 46 |
| C4 | RUN2217 | P1 | I | 31 | STI-13 | Reunion | 2014 | *Solanum lycopersicum* | 23 | 20 | 22 | 22 | 20 | 29 | 43 |
| C6 | RUN2267 | TG5C | IIB | 1 | STII-29 | Madagascar | 2013 | *Solanum tuberosum* | 21 | 26 | 23 | 19 | 19 | 30 | 50 |
| C6 | RUN2276 | MG6 | III | 19 | STIII-39 | Madagascar | 2013 | *Solanum tuberosum* | 3 | 13 | 11 | 11 | 24 | 7 | 11 |
| C6 | RUN2310 | MG44 | III | 60 | STIII-35 | Madagascar | 2013 | *Phaseolus vulgaris* | 25 | 22 | 13 | 11 | 6 | 64 | 73 |
| C6 | RUN2340 | MG78 | III | 58 | STIII-33 | Madagascar | 2013 | *Solanum tuberosum* | 24 | 22 | 13 | 11 | 6 | 65 | 72 |
| C6 | RUN2344 | MG82 | III | 58 | STIII-33 | Madagascar | 2013 | *Solanum tuberosum* | 24 | 22 | 13 | 11 | 6 | 65 | 72 |
| C6 | RUN2510 | MG275 | III | 60 | STIII-37 | Madagascar | 2013 | *Solanum tuberosum* | 25 | 22 | 25 | 11 | 4 | 7 | 73 |
| C6 | RUN2658 | MG350 | IIB | 1 | STII-29 | Madagascar | 2013 | *Solanum tuberosum* | 21 | 26 | 23 | 19 | 19 | 30 | 50 |
| C6 | RUN2677 | MG392 | IIB | 1 | STII-29 | Madagascar | 2013 | *Solanum tuberosum* | 21 | 26 | 23 | 19 | 19 | 30 | 50 |
| C6 | RUN2706 | MG533 | III | 19 | STIII-40 | Madagascar | 2013 | *Solanum tuberosum* | 3 | 22 | 12 | 11 | 25 | 7 | 11 |
| C6 | RUN2717 | MG546 | III | 60 | STIII-44 | Madagascar | 2013 | *Solanum tuberosum* | 3 | 27 | 11 | 11 | 24 | 7 | 73 |
| C6 | RUN2722 | MG551 | III | 60 | STIII-41 | Madagascar | 2013 | *Solanum tuberosum* | 3 | 22 | 13 | 11 | 4 | 7 | 73 |
| C6 | RUN2735 | MG564 | IIB | 1 | STII-29 | Madagascar | 2013 | *Solanum tuberosum* | 21 | 26 | 23 | 19 | 19 | 30 | 50 |
| C6 | RUN2786 | MG621 | III | 19 | STIII-46 | Madagascar | 2013 | *Solanum tuberosum* | 3 | 22 | 13 | 11 | 4 | 7 | 11 |
| C4 | RUN2996 | P6 | IIB | 1 | STII-29 | Reunion | 2014 | *Solanum lycopersicum* | 21 | 26 | 23 | 19 | 19 | 30 | 50 |
| C6 | RUN3083 | 86 | I | 18 | STI-12 | Madagascar | 2013 | *Solanum lycopersicum* | 22 | 25 | 22 | 21 | 17 | 29 | 42 |
| C6 | RUN3148 | 166 | I | 33 | STI-9 | Madagascar | 2013 | *Solanum tuberosum* | 23 | 24 | 22 | 22 | 18 | 26 | 76 |
| C6 | RUN3216 | 257 | I | 33 | STI-9 | Madagascar | 2013 | *Solanum lycopersicum* | 23 | 24 | 22 | 22 | 18 | 26 | 76 |
| C6 | RUN3230 | 272 | III | 60 | STIII-36 | Madagascar | 2013 | *Solanum tuberosum* | 25 | 22 | 13 | 11 | 6 | 7 | 73 |
| C6 | RUN3277 | 368 | I | 18 | STI-12 | Madagascar | 2013 | *Solanum aethiopicum* | 22 | 25 | 22 | 21 | 17 | 29 | 42 |
| C6 | RUN3304 | 410 | III | 59 | STIII-34 | Madagascar | 2013 | *Solanum tuberosum* | 25 | 22 | 13 | 11 | 26 | 64 | 74 |
| C6 | RUN3321 | 434 | III | 60 | STIII-37 | Madagascar | 2013 | *Solanum tuberosum* | 25 | 22 | 25 | 11 | 4 | 7 | 73 |
| C8 | RUN3489 | LB107 | I | 31 | STI-13 | Seychelles | 2005 | *Solanum melongena* | 23 | 20 | 22 | 22 | 20 | 29 | 43 |
| C8 | RUN3490 | JT675 | I | 31 | STI-13 | Seychelles | 1998 | *Solanum lycopersicum* | 23 | 20 | 22 | 22 | 20 | 29 | 43 |
| C7 | RUN3491 | LB217 | I | 31 | STI-13 | Comoros | 2005 | *Solanum lycopersicum* | 23 | 20 | 22 | 22 | 20 | 29 | 43 |
| C7 | RUN3492 | LB218 | I | 31 | STI-13 | Comoros | 2005 | *Phaseolus vulgaris* | 23 | 20 | 22 | 22 | 20 | 29 | 43 |
| C7 | RUN3493 | LB219 | I | 31 | STI-13 | Comoros | 2005 | *Solanum tuberosum* | 23 | 20 | 22 | 22 | 20 | 29 | 43 |
| C7 | RUN3494 | LB220 | I | 31 | STI-13 | Comoros | 2005 | *Solanum tuberosum* | 23 | 20 | 22 | 22 | 20 | 29 | 43 |
| C7 | RUN3495 | LB221 | I | 31 | STI-13 | Comoros | 2005 | *Solanum lycopersicum* | 23 | 20 | 22 | 22 | 20 | 29 | 43 |
| C5 | RUN3501 | JS940 | I | 33 | STI-9 | Mauritius | 1957 | *Vicia faba* | 23 | 24 | 22 | 22 | 18 | 26 | 76 |
| C6 | RUN3503 | 66 | III | 19 | STIII-43 | Madagascar | 2013 | *Solanum tuberosum* | 3 | 22 | 26 | 11 | 4 | 7 | 11 |
| C5 | RUN3505 | JS944 | I | 33 | STI-9 | Mauritius | 1957 | *Brassica oleracea* | 23 | 24 | 22 | 22 | 18 | 26 | 76 |
| C5 | RUN3510 | JS954 | I | 31 | STI-13 | Mauritius | 1957 | *Casuarina equisetifolia* | 23 | 20 | 22 | 22 | 20 | 29 | 43 |
| C5 | RUN3513 | JS955 | I | 33 | STI-9 | Mauritius | 1957 | *Dahlia sp.* | 23 | 24 | 22 | 22 | 18 | 26 | 76 |
| C2 | RUN3560 | Rs-TH-BG-1 | I | 15 | STI-10 | Thailand | 2009 | *Momordica charantia* | 19 | 18 | 24 | 22 | 22 | 27 | 47 |
| C2 | RUN3563 | Rs-TH-EGP-2 | I | 15 | STI-10 | Thailand | 2007 | *Solanum melongena* | 19 | 18 | 24 | 22 | 22 | 27 | 47 |
| C2 | RUN3564 | Rs-TH-MA-1 | I | 15 | STI-10 | Thailand | 2011 | *Tagetes sp.* | 19 | 18 | 24 | 22 | 22 | 27 | 47 |
| C4 | RUN3573 | BMa-D16 | I | 33 | STI-9 | Reunion | 2014 | *Solanum lycopersicum* | 23 | 24 | 22 | 22 | 18 | 26 | 76 |
| C4 | RUN3611 | PR1.4b(1) | IIB | 1 | STII-29 | Reunion | 2014 | *Solanum tuberosum* | 21 | 26 | 23 | 19 | 19 | 30 | 50 |
| C2 | RUN3633 | 42796 | I | 31 | STI-13 | Benin | 2014 | *Solanum macrocarpon* | 23 | 20 | 22 | 22 | 20 | 29 | 43 |
| C4 | RUN3666 | T2 | IIB | 1 | STII-29 | Reunion | 2014 | *Solanum lycopersicum* | 21 | 26 | 23 | 19 | 19 | 30 | 50 |
| C4 | RUN3691 | Chamand | I | 31 | STI-13 | Reunion | 2014 | *Solanum lycopersicum* | 23 | 20 | 22 | 22 | 20 | 29 | 43 |
| C4 | RUN3699 | Dom 8.7 | IIB | 1 | STII-29 | Reunion | 2015 | *Solanum tuberosum* | 21 | 26 | 23 | 19 | 19 | 30 | 50 |
| C4 | RUN3711 | 2,1 | I | 31 | STI-13 | Reunion | 2014 | *Pelargonium cv. rosé* | 23 | 20 | 22 | 22 | 20 | 29 | 43 |
| C4 | RUN3714 | N'1 (7) | III | 19 | STIII-46 | Reunion | 2014 | *Pelargonium cv. rosé* | 3 | 22 | 13 | 11 | 4 | 7 | 11 |
| C4 | RUN3732 | 6,1 | III | 19 | STIII-46 | Reunion | 2014 | Weeds | 3 | 22 | 13 | 11 | 4 | 7 | 11 |
| C4 | RUN3733 | 6,2 | III | 19 | STIII-46 | Reunion | 2014 | *Solanum lycopersicum* | 3 | 22 | 13 | 11 | 4 | 7 | 11 |
| C4 | RUN3740 | 8,1 | III | 19 | STIII-47 | Reunion | 2014 | *Pelargonium cv. rosé* | 23 | 22 | 13 | 11 | 4 | 7 | 11 |
| C4 | RUN3743 | Tony 36 | I | 31 | STI-13 | Reunion | 2015 | *Solanum melongena* | 23 | 20 | 22 | 22 | 20 | 29 | 43 |
| C4 | RUN3744 | 9,2 | III | 19 | STIII-46 | Reunion | 2014 | *Pelargonium cv. rosé* | 3 | 22 | 13 | 11 | 4 | 7 | 11 |
| C4 | RUN3756 | Bruno1 | I | 31 | STI-13 | Reunion | 2014 | *Solanum melongena* | 23 | 20 | 22 | 22 | 20 | 29 | 43 |
| C4 | RUN3770 | RAS1 | I | 31 | STI-13 | Reunion | 2015 | *Solanum melongena* | 23 | 20 | 22 | 22 | 20 | 29 | 43 |
| C4 | RUN3812 | S1T1 | IIA | 36 | STII-7 | Reunion | 2015 | *Solanum lycopersicum* | 18 | 17 | 19 | 17 | 16 | 23 | 49 |
| C4 | RUN3815 | JCB3 | I | 31 | STI-13 | Reunion | 2015 | *Solanum melongena* | 23 | 20 | 22 | 22 | 20 | 29 | 43 |
| C4 | RUN3841 | O1 | I | 31 | STI-13 | Reunion | 2015 | *Pelargonium cv. rosé* | 23 | 20 | 22 | 22 | 20 | 29 | 43 |
| C4 | RUN3856 | DR1G | I | 31 | STI-13 | Reunion | 2015 | *Pelargonium cv. rosé* | 23 | 20 | 22 | 22 | 20 | 29 | 43 |
| C4 | RUN3862 | SG14 | III | 19 | STIII-46 | Reunion | 2015 | *Pelargonium cv. rosé* | 3 | 22 | 13 | 11 | 4 | 7 | 11 |
| C4 | RUN3869 | SG14 | I | 31 | STI-13 | Reunion | 2015 | *Pelargonium cv. rosé* | 23 | 20 | 22 | 22 | 20 | 29 | 43 |
| C4 | RUN3884 | EG13 | I | 31 | STI-13 | Reunion | 2015 | *Pelargonium cv. rosé* | 23 | 20 | 22 | 22 | 20 | 29 | 43 |
| C4 | RUN3893 | LJCT2 | I | 31 | STI-13 | Reunion | 2015 | *Solanum lycopersicum* | 23 | 20 | 22 | 22 | 20 | 29 | 43 |
| C4 | RUN3928 | EGCG1 | I | 31 | STI-13 | Reunion | 2015 | *Pelargonium cv. rosé* | 23 | 20 | 22 | 22 | 20 | 29 | 43 |
| C4 | RUN4047 | HGT1 | I | 31 | STI-13 | Reunion | 2015 | *Solanum lycopersicum* | 23 | 20 | 22 | 22 | 20 | 29 | 43 |
| C4 | RUN4076 | MFbrede1 | I | 31 | STI-13 | Reunion | 2015 | Weeds | 23 | 20 | 22 | 22 | 20 | 29 | 43 |
| C4 | RUN4079 | MFT8 | I | 31 | STI-13 | Reunion | 2015 | *Solanum lycopersicum* | 23 | 20 | 22 | 22 | 20 | 29 | 43 |
| C4 | RUN4082 | GJBG4 | I | 31 | STI-13 | Reunion | 2015 | *Pelargonium cv. rosé* | 23 | 20 | 22 | 22 | 20 | 29 | 43 |
| C4 | RUN4105 | HRGP1 | I | 31 | STI-13 | Reunion | 2015 | *Capsicum annuum* | 23 | 20 | 22 | 22 | 20 | 29 | 43 |
| C4 | RUN4112 | HRA1 | I | 31 | STI-13 | Reunion | 2015 | *Solanum melongena* | 23 | 20 | 22 | 22 | 20 | 29 | 43 |
| C4 | RUN4170 | MNT1 | I | 31 | STI-13 | Reunion | 2015 | *Solanum lycopersicum* | 23 | 20 | 22 | 22 | 20 | 29 | 43 |
| C4 | RUN4196 | MF far 1 | I | 31 | STI-13 | Reunion | 2015 | *Solanum lycopersicum* | 23 | 20 | 22 | 22 | 20 | 29 | 43 |
| C4 | RUN4210 | RE 1.3.2 | I | 31 | STI-13 | Reunion | 2015 | *Solanum lycopersicum* | 23 | 20 | 22 | 22 | 20 | 29 | 43 |
| C4 | RUN4257 | Haricot1 | I | 31 | STI-13 | Reunion | 2015 | *Phaseolus vulgaris* | 23 | 20 | 22 | 22 | 20 | 29 | 43 |
| C4 | RUN4263 | Adv2 | I | 31 | STI-13 | Reunion | 2015 | Weeds | 23 | 20 | 22 | 22 | 20 | 29 | 43 |
| C8 | RUN4292 | 44 | I | 31 | STI-13 | Seychelles | 2015 | *Solanum melongena* | 23 | 20 | 22 | 22 | 20 | 29 | 43 |
| C8 | RUN4303 | 122 | I | 31 | STI-13 | Seychelles | 2015 | *Capsicum annuum* | 23 | 20 | 22 | 22 | 20 | 29 | 43 |
| C8 | RUN4308 | 145 | I | 31 | STI-13 | Seychelles | 2015 | *Solanum melongena* | 23 | 20 | 22 | 22 | 20 | 29 | 43 |
| C8 | RUN4311 | 172 | I | 31 | STI-13 | Seychelles | 2015 | *Solanum lycopersicum* | 23 | 20 | 22 | 22 | 20 | 29 | 43 |
| C8 | RUN4315 | 214 | I | 31 | STI-13 | Seychelles | 2015 | *Solanum melongena* | 23 | 20 | 22 | 22 | 20 | 29 | 43 |
| C8 | RUN4325 | 240 | I | 31 | STI-13 | Seychelles | 2015 | Weeds | 23 | 20 | 22 | 22 | 20 | 29 | 43 |
| C8 | RUN4326 | 241 | I | 31 | STI-13 | Seychelles | 2015 | *Solanum lycopersicum* | 23 | 20 | 22 | 22 | 20 | 29 | 43 |
| C8 | RUN4337 | 87 | I | 31 | STI-13 | Seychelles | 2015 | *Solanum melongena* | 23 | 20 | 22 | 22 | 20 | 29 | 43 |
| C5 | RUN4376 | 3 | I | 31 | STI-13 | Mauritius | 2015 | *Solanum melongena* | 23 | 20 | 22 | 22 | 20 | 29 | 43 |
| C5 | RUN4392 | 43 | I | 15 | STI-11 | Mauritius | 2015 | *Solanum lycopersicum* | 23 | 19 | 20 | 20 | 22 | 29 | 46 |
| C5 | RUN4393 | 46 K | I | 15 | STI-11 | Mauritius | 2015 | *Capsicum annuum* | 23 | 19 | 20 | 20 | 22 | 29 | 46 |
| C5 | RUN4400 | 83 | I | 33 | STI-9 | Mauritius | 2015 | *Solanum lycopersicum* | 23 | 24 | 22 | 22 | 18 | 26 | 76 |
| C5 | RUN4406 | 89 amaranthe | I | 33 | STI-9 | Mauritius | 2015 | Weeds | 23 | 24 | 22 | 22 | 18 | 26 | 76 |
| C5 | RUN4407 | 90 | I | 15 | STI-11 | Mauritius | 2015 | *Solanum lycopersicum* | 23 | 19 | 20 | 20 | 22 | 29 | 46 |
| C4 | RUN4438 | Pepino 7 | I | 31 | STI-13 | Reunion | 2015 | *Solanum muricatum* | 23 | 20 | 22 | 22 | 20 | 29 | 43 |
| C4 | RUN4450 | GP1 | I | 31 | STI-13 | Reunion | 2015 | *Capsicum annuum* | 23 | 20 | 22 | 22 | 20 | 29 | 43 |
| C4 | RUN4478 | BM3 | IIB | 1 | STII-29 | Reunion | 2015 | *Solanum nigrum* | 21 | 26 | 23 | 19 | 19 | 30 | 50 |
| C4 | RUN4509 | Tu2P3 | IIB | 1 | STII-29 | Reunion | 2015 | *Solanum tuberosum* | 21 | 26 | 23 | 19 | 19 | 30 | 50 |
| C5 | RUN4584 | 1,3 | I | 18 | STI-12 | Mauritius | 2015 | *Solanum tuberosum* | 22 | 25 | 22 | 21 | 17 | 29 | 42 |
| C5 | RUN4603 | 2,1 | I | 33 | STI-9 | Mauritius | 2015 | *Solanum tuberosum* | 23 | 24 | 22 | 22 | 18 | 26 | 76 |
| C5 | RUN4606 | 2,4 | IV | 10 | STIV-12 | Mauritius | 2015 | *Solanum tuberosum* | 20 | 21 | 21 | 18 | 21 | 28 | 51 |
| C5 | RUN4645 | 3,4 | I | 18 | STI-12 | Mauritius | 2015 | *Solanum sp.* | 22 | 25 | 22 | 21 | 17 | 29 | 42 |
| C5 | RUN4664 | 4,29 | I | 15 | STI-11 | Mauritius | 2015 | *Solanum tuberosum* | 23 | 19 | 20 | 20 | 22 | 29 | 46 |
| C5 | RUN4680 | 11,1 | I | 33 | STI-9 | Mauritius | 2015 | *Solanum tuberosum* | 23 | 24 | 22 | 22 | 18 | 26 | 76 |
| C5 | RUN4847 | 2,7 | IV | 10 | STIV-12 | Mauritius | 2015 | *Solanum tuberosum* | 20 | 21 | 21 | 18 | 21 | 28 | 51 |
| C5 | RUN4899 | 20,1 | I | 31 | STI-13 | Mauritius | 2015 | *Solanum tuberosum* | 23 | 20 | 22 | 22 | 20 | 29 | 43 |
| C5 | RUN4953 | 22,2 | I | 15 | STI-11 | Mauritius | 2015 | *Solanum tuberosum* | 23 | 19 | 20 | 20 | 22 | 29 | 46 |
| C5 | RUN5007 | 23,1 | I | 15 | STI-11 | Mauritius | 2015 | *Solanum tuberosum* | 23 | 19 | 20 | 20 | 22 | 29 | 46 |
| C5 | RUN5135 | 25,1 | I | 18 | STI-12 | Mauritius | 2015 | *Cyphostemma mappia* | 22 | 25 | 22 | 21 | 17 | 29 | 42 |
| C5 | RUN5137 | 8,2 | I | 15 | STI-11 | Mauritius | 2015 | *Capsicum frutescens* | 23 | 19 | 20 | 20 | 22 | 29 | 46 |
| C5 | RUN5138 | 6,27 | I | 33 | STI-9 | Mauritius | 2015 | *Solanum tuberosum* | 23 | 24 | 22 | 22 | 18 | 26 | 76 |
| C5 | RUN5163 | 19,3 | I | 31 | STI-13 | Mauritius | 2015 | *Solanum tuberosum* | 23 | 20 | 22 | 22 | 20 | 29 | 43 |
| C4 | RUN5253 | 1.31T | I | 31 | STI-13 | Reunion | 2015 | *Solanum lycopersicum* | 23 | 20 | 22 | 22 | 20 | 29 | 43 |
| C4 | RUN5274 | 2,32 | III | 19 | STIII-46 | Reunion | 2015 | *Solanum tuberosum* | 3 | 22 | 13 | 11 | 4 | 7 | 11 |
| C4 | RUN5277 | 2,28 | I | 31 | STI-13 | Reunion | 2015 | *Solanum tuberosum* | 23 | 20 | 22 | 22 | 20 | 29 | 43 |
| C5 | RUN5331 | M3H1.1 | I | 14 | STI-8 | Mauritius | 2016 | *Phaseolus vulgaris* | 23 | 18 | 22 | 22 | 22 | 25 | 44 |
| C5 | RUN5385 | M3A7.63 | I | 33 | STI-9 | Mauritius | 2016 | *Anthurium andreanum* | 23 | 24 | 22 | 22 | 18 | 26 | 76 |
| C10 | RUN5419 | M3T10.127 | I | 31 | STI-13 | Rodrigues | 2016 | *Solanum lycopersicum* | 23 | 20 | 22 | 22 | 20 | 29 | 43 |
| C10 | RUN5445 | M3Pi9.107 | I | 33 | STI-9 | Rodrigues | 2016 | *Capsicum frutescens* | 23 | 24 | 22 | 22 | 18 | 26 | 76 |
| C5 | RUN5453 | M3T4.59 | I | 31 | STI-13 | Mauritius | 2016 | *Solanum lycopersicum* | 23 | 20 | 22 | 22 | 20 | 29 | 43 |
| C5 | RUN5454 | M3An5.60 | I | 33 | STI-9 | Mauritius | 2016 | *Anthurium andreanum* | 23 | 24 | 22 | 22 | 18 | 26 | 76 |
| C5 | RUN5455 | M3An6.61 | I | 33 | STI-9 | Mauritius | 2016 | *Anthurium andreanum* | 23 | 24 | 22 | 22 | 18 | 26 | 76 |
| C10 | RUN5456 | M3Pi16.175 | I | 33 | STI-9 | Rodrigues | 2016 | *Capsicum frutescens* | 23 | 24 | 22 | 22 | 18 | 26 | 76 |
| C7 | RUN5479 | BDMT1 | I | 31 | STI-13 | Comoros | 2016 | *Solanum lycopersicum* | 23 | 20 | 22 | 22 | 20 | 29 | 43 |
| C7 | RUN5480 | BA2-2-PT3 | I | 31 | STI-13 | Comoros | 2016 | *Solanum tuberosum* | 23 | 20 | 22 | 22 | 20 | 29 | 43 |
| C7 | RUN5481 | KOD2A2 | I | 31 | STI-13 | Comoros | 2016 | *Solanum melongena* | 23 | 20 | 22 | 22 | 20 | 29 | 43 |
| C7 | RUN5482 | NDRB2T1 | I | 31 | STI-13 | Comoros | 2016 | *Solanum lycopersicum* | 23 | 20 | 22 | 22 | 20 | 29 | 43 |
| C7 | RUN5485 | NIM3T1 | I | 31 | STI-13 | Comoros | 2016 | *Solanum lycopersicum* | 23 | 20 | 22 | 22 | 20 | 29 | 43 |
| C7 | RUN5489 | WAL2T1 | I | 31 | STI-13 | Comoros | 2016 | *Solanum lycopersicum* | 23 | 20 | 22 | 22 | 20 | 29 | 43 |
| C7 | RUN5495 | NIM1T1 | I | 31 | STI-13 | Comoros | 2016 | *Solanum lycopersicum* | 23 | 20 | 22 | 22 | 20 | 29 | 43 |
| C7 | RUN5508 | NDRB1T1 | I | 31 | STI-13 | Comoros | 2016 | *Solanum lycopersicum* | 23 | 20 | 22 | 22 | 20 | 29 | 43 |
| C7 | RUN5513 | LINT1 | I | 31 | STI-13 | Comoros | 2016 | *Solanum lycopersicum* | 23 | 20 | 22 | 22 | 20 | 29 | 43 |
| C7 | RUN5514 | NIM2T1 | I | 31 | STI-13 | Comoros | 2016 | *Solanum lycopersicum* | 23 | 20 | 22 | 22 | 20 | 29 | 43 |
| C7 | RUN5516 | MRMA1 | I | 31 | STI-13 | Comoros | 2016 | *Solanum melongena* | 23 | 20 | 22 | 22 | 20 | 29 | 43 |
| C7 | RUN5521 | OUAT1 | I | 31 | STI-13 | Comoros | 2016 | *Solanum lycopersicum* | 23 | 20 | 22 | 22 | 20 | 29 | 43 |
| C7 | RUN5525 | PATT3 | I | 31 | STI-13 | Comoros | 2016 | *Solanum lycopersicum* | 23 | 20 | 22 | 22 | 20 | 29 | 43 |
