# Supplementary material for: Genetic Diversity of the Ralstonia solanacearum Species Complex in the Southwest Indian Ocean Islands
Source: Front Plant Sci. 2017 Dec 19;8:2139. doi: 10.3389/fpls.2017.02139 (PMC5742265; doi:10.3389/fpls.2017.02139)
Supplement: Table S2 — Description of MLST loci, corresponding oligonucleotide primers, and PCR conditions. [file Table2.DOCX]

| **LOCUS** | **REFERENCE NAME OF THE LOCUS** | **GENE SIZE (BP)** | **GMI1000 POSITION** | **OLIGOPEPTIDE PRIMER (5' TO 3')** | **ANNEALING TEMPERATURE (°C)** | **AMPLICON SIZE (BP)** | **SIZE OF THE ANALYSED SEQUENCE** | **START SEQUENCE 5'** | **END SEQUENCE 3'** | **REFERENCE** |
| --- | --- | --- | --- | --- | --- | --- | --- | --- | --- | --- |
| CHROMOSOME: HOUSEKEEPING GENES | | | | | | | | | | |
| ***gdhA*** | glutamate dehydrogenase | 1302 | 515594-516896 | F : GAGAAATCCTGCACCCACTCGAAA | 55 | 639 | 603 | CGAAAT/ CGAAGT | GCGCGT | Wicker et al., 2007 |
|  |  |  |  | R : GCCGGACGTGAACACCAAC |  |  |  |  |  |  |
| ***gyrB*** | DNA gyrase | 2529 | 3710650-3713179 | F : ATCCACGTCACCATCCACAC | 68 | 460 | 393 | ATTCCG/ ATCCCG | CTCTCC | Ravelomanantsoa et al., 2016 |
|  |  |  |  | R : GGATGCGCTTGGAGAGGAT |  |  |  |  |  |  |
| ***rplB*** | 50S ribosomal | 831 | 3237414-3238245 | F : CCGCTCGATGGTGAAGGTCGT | 62 | 766 | 654 | GTCAAT/ GTCAAC | GGTGAA | Wicker et al., 2007 |
|  |  |  |  | R : CATGCTGGTCGTGCGCTTGTTG |  |  |  |  |  |  |
| ***leuS*** | Leucyl-tRNA synthetase | 2634 | 2953327-2955961 | F : GGTCGAACAGCAGGCGCAGCAA | 68 | 793 | 723 | GATGCC/GCAGCC | ACGCGT/ ACGCGC/ ACGCGG | Wicker et al., 2007 |
|  |  |  |  | R : GGCGCAGAAGGTCACGCCCA |  |  |  |  |  |  |
| ***adk*** | Adenylate kinase | 669 | 2740594-2741263 | F : CCCAGCCGGAGTAGTAGTCC | 62 | 545 | 468 | GGACAG | CGTACC | Castillo & Greenberg, 2007 |
|  |  |  |  | R : TCTGTTGGGCGCACCCGGC |  |  |  |  |  |  |
| ***mutS*** | methyl-directed DNA mismatch repair protein | 2649 | 1207216-1209865 | F : GCTGATCACCGGCCCGAACAT | 67 | 758 | 681 | GGGGTC/ CGGGTC | ACCGCC | Prior & Fegan, 2005 |
|  |  |  |  | R : ACAGCGCCTTGAGCCGGTACA |  |  |  |  |  |  |
| MEGAPLASMIDE: VIRULENCE-ASSOCIATED GENES | | | | | | | | | | |
| ***egl*** | Endoglucanase precursor | 1311 | 188552-189863 | Endo-F : ATGCATGCCGCTGGTCGCCGC | 70 | 844 | 710 | ACGGCG/ ACGGTG/ ACGGAC | CAGTGG | Poussier et al., 2000 |
|  |  |  |  | Endo-R : GCGTTGCCCGGCACGAACACC |  |  |  |  |  |  |
