# Supplementary material for: Genetic Diversity of the Ralstonia solanacearum Species Complex in the Southwest Indian Ocean Islands
Source: Front Plant Sci. 2017 Dec 19;8:2139. doi: 10.3389/fpls.2017.02139 (PMC5742265; doi:10.3389/fpls.2017.02139)
Supplement: Table S3 — Genetic properties of the loci used in MLSA for each phylotype of the SWIO (C1) and the worldwide (C2) RSSC collections. [file Table3.DOCX]

| **Collection** | **Locus** | **Length (bp)** | **Hap** | **S** | **Hd** | **G+C** | **Θπ** | **Tajima's D** | **Fu & Li's D** | **Fu's F** |
| --- | --- | --- | --- | --- | --- | --- | --- | --- | --- | --- |
| **C1 (n=145)** |  |  |  |  |  |  |  |  |  |  |
| **Phylotype I (n=104)** | *gdhA* | 603 | 2 | 1 | 0.127 | 0.665 | 0.000 | -0.385 | 0.49 | -0.090 |
|  | *gyrB* | 391 | 6 | 21 | 0.518 | 0.646 | 0.013 | 0.477 | 1.369 | 8.61 |
|  | *rplB* | 654 | 2 | 1 | 0.143 | 0.648 | 0.000 | -0.287 | 0.49 | 0.077 |
|  | *leuS* | 723 | 3 | 2 | 0.244 | 0.647 | 0.000 | -0.518 | 0.682 | -0.615 |
|  | *adK* | 468 | 4 | 5 | 0.513 | 0.646 | 0.003 | 1.085 | 1.034 | 2.864 |
|  | *mutS* | 693 | 5 | 34 | 0.327 | 0.718 | 0.001 | -2.636*** | -7.765** | 0.258 |
|  | *egl* | 713 | 7 | 14 | 0.530 | 0.688 | 0.004 | -0.248 | -0.166 | 2.183 |
|  | Concatenate | 4245 | 9 | 78 | 0.544 | 0.668 | 0.002 | -0.105 | -3.194* | 13.906 |
|  |  |  |  |  |  |  |  |  |  |  |
| **Phylotype II (n=13)** | *gdhA* | 603 | 2 | 6 | 0.154 | 0.662 | 0.016 | -1.930* | -2.421* | 2.300 |
|  | *gyrB* | 391 | 3 | 6 | 0.295 | 0.647 | 0.002 | -1.930* | -2.609* | 0.610 |
|  | *rplB* | 654 | 2 | 12 | 0.154 | 0.646 | 0.003 | -2.134** | -2.732** | 4.223 |
|  | *leuS* | 723 | 2 | 10 | 0.154 | 0.640 | 0.002 | -2.088** | -2.660** | 3.639 |
|  | *adK* | 468 | 2 | 3 | 0.154 | 0.646 | 0.001 | -1.652 | -2.026 | 0.976 |
|  | *mutS* | 693 | 2 | 18 | 0.154 | 0.706 | 0.004 | -2.218** | -2.865** | 5.706 |
|  | *egl* | 713 | 2 | 28 | 0.154 | 0.669 | 0.006 | -2.284*** | -2.973** | 7.898 |
|  | Concatenate | 4245 | 3 | 83 | 0.295 | 0.662 | 0.003 | -2.371*** | -3.120** | 11.612 |
|  |  |  |  |  |  |  |  |  |  |  |
| **Phylotype III (n=26)** | *gdhA* | 603 | 4 | 10 | 0.495 | 0.660 | 0.003 | -0.725 | -1.690 | 2.283 |
|  | *gyrB* | 391 | 5 | 6 | 0.462 | 0.642 | 0.002 | -1.228 | -1.023 | -1.008 |
|  | *rplB* | 654 | 5 | 9 | 0.514 | 0.645 | 0.002 | -1.805 | -1.983 | -0.705 |
|  | *leuS* | 723 | 1 | 0 | 0.000 | 0.650 | 0.000 | nd | nd | nd |
|  | *adK* | 468 | 7 | 19 | 0.643 | 0.654 | 0.006 | -1.404 | -2.415 | 0.595 |
|  | *mutS* | 693 | 3 | 13 | 0.283 | 0.710 | 0.003 | -1.364 | 1.491* | 3.766 |
|  | *egl* | 713 | 4 | 23 | 0.532 | 0.684 | 0.013 | 2.042* | 1.646** | 11.889 |
|  | Concatenate | 4245 | 16 | 80 | 0.920 | 0.666 | 0.004 | -0.463 | -0.523 | 0.732 |
|  |  |  |  |  |  |  |  |  |  |  |
| **Phylotype IV (n=2)** | *gdhA* | 603 | 1 | 0 | 0.000 | 0.654 | 0.000 | nd | nd | nd |
|  | *gyrB* | 391 | 1 | 0 | 0.000 | 0.657 | 0.000 | nd | nd | nd |
|  | *rplB* | 654 | 1 | 0 | 0.000 | 0.650 | 0.000 | nd | nd | nd |
|  | *leuS* | 723 | 1 | 0 | 0.000 | 0.639 | 0.000 | nd | nd | nd |
|  | *adK* | 468 | 1 | 0 | 0.000 | 0.654 | 0.000 | nd | nd | nd |
|  | *mutS* | 693 | 1 | 0 | 0.000 | 0.711 | 0.000 | nd | nd | nd |
|  | *egl* | 713 | 1 | 0 | 0.000 | 0.671 | 0.000 | nd | nd | nd |
|  | Concatenate | 4245 | 1 | 0 | 0.000 | 0.663 | 0.000 | nd | nd | nd |
|  |  |  |  |  |  |  |  |  |  |  |
| **C2 (n=90)** |  |  |  |  |  |  |  |  |  |  |
| **Phylotype I (n=26)** | *gdhA* | 603 | 6 | 8 | 0.671 | 0.665 | 0.002 | -1.354 | -1.11 | -1.320 |
|  | *gyrB* | 391 | 15 | 25 | 0.935 | 0.653 | 0.024 | 1.01 | 0.804 | -1.190 |
|  | *rplB* | 654 | 5 | 6 | 0.726 | 0.647 | 0.002 | 0.104 | 0.476 | 0.505 |
|  | *leuS* | 723 | 6 | 5 | 0.551 | 0.647 | 0.001 | -1.470 | -2.241 | -3.330 |
|  | *adK* | 468 | 12 | 16 | 0.871 | 0.650 | 0.009 | -0.618 | 0.055 | -2.283 |
|  | *mutS* | 693 | 9 | 15 | 0.705 | 0.718 | 0.003 | -1.928* | -2.218 | -3.090 |
|  | *egl* | 713 | 17 | 20 | 0.957 | 0.689 | 0.007 | -0.434 | -0.458 | -7.418 |
|  | Concatenate | 4245 | 21 | 95 | 0.972 | 0.669 | 0.006 | -0.473 | -0.505 | -2.139 |
|  |  |  |  |  |  |  |  |  |  |  |
| **Phylotype II (n=41)** | *gdhA* | 603 | 12 | 21 | 0.878 | 0.659 | 0.009 | 0.345 | 0.01 | 0.328 |
|  | *gyrB* | 391 | 25 | 27 | 0.967 | 0.648 | 0.015 | -0.449 | -0.606 | -11.743 |
|  | *rplB* | 654 | 12 | 50 | 0.850 | 0.645 | 0.013 | -1.013 | -2.326 | 2.473 |
|  | *leuS* | 723 | 18 | 34 | 0.929 | 0.644 | 0.011 | -0.008 | 0.084 | -1.442 |
|  | *adK* | 468 | 26 | 24 | 0.956 | 0.649 | 0.010 | -0.793 | -0.498 | -16.235 |
|  | *mutS* | 693 | 18 | 38 | 0.927 | 0.705 | 0.016 | 0.672 | -0.330 | 0.021 |
|  | *egl* | 713 | 27 | 80 | 0.965 | 0.675 | 0.026 | -0.149 | -0.547 | -2.760 |
|  | Concatenate | 4245 | 40 | 274 | 0.999 | 0.662 | 0.015 | -0.238 | -0.797 | -8.415 |
|  |  |  |  |  |  |  |  |  |  |  |
| **Phylotype III (n=12)** | *gdhA* | 603 | 12 | 24 | 1.000 | 0.660 | 0.010 | -1.104 | -1.393 | -7.029 |
|  | *gyrB* | 391 | 10 | 16 | 0.955 | 0.641 | 0.013 | -0.691 | -0.354 | -3.630 |
|  | *rplB* | 654 | 12 | 53 | 1.000 | 0.644 | 0.016 | -1.914* | -2.230* | -4.776 |
|  | *leuS* | 723 | 12 | 27 | 1.000 | 0.650 | 0.008 | -1.495 | -1.667 | -7.254 |
|  | *adK* | 468 | 7 | 6 | 0.864 | 0.658 | 0.004 | -0.353 | -0.527 | -2.891 |
|  | *mutS* | 693 | 12 | 32 | 1.000 | 0.714 | 0.014 | -0.544 | -0.616 | -5.219 |
|  | *egl* | 713 | 12 | 38 | 1.000 | 0.692 | 0.017 | -0.486 | -0.623 | -4.320 |
|  | Concatenate | 4245 | 12 | 196 | 1.000 | 0.668 | 0.012 | -1.145 | -1.318 | -0.864 |
|  |  |  |  |  |  |  |  |  |  |  |
| **Phylotype IV (n=11)** | *gdhA* | 603 | 7 | 41 | 0.873 | 0.654 | 0.020 | -0.935 | -0.538 | 1.884 |
|  | *gyrB* | 391 | 11 | 41 | 1.000 | 0.651 | 0.033 | -0.504 | -0.434 | -3.416 |
|  | *rplB* | 654 | 8 | 52 | 0.891 | 0.650 | 0.027 | -0.357 | 0.052 | 1.589 |
|  | *leuS* | 723 | 8 | 35 | 0.927 | 0.641 | 0.017 | 0.034 | 0.018 | 0.699 |
|  | *adK* | 468 | 10 | 24 | 0.982 | 0.654 | 0.017 | -0.582 | -0.291 | -2.857 |
|  | *mutS* | 693 | 8 | 47 | 0.891 | 0.713 | 0.020 | -0.811 | -0.585 | 1.036 |
|  | *egl* | 713 | 8 | 68 | 0.927 | 0.672 | 0.037 | 0.401 | 0.206 | 2.635 |
|  | Concatenate | 4245 | 11 | 308 | 1.000 | 0.663 | 0.024 | -0.3471 | -0.196 | 0.379 |
|  |  |  |  |  |  |  |  |  |  |  |
| Significance: *P<0.05; ** P<0.02; *** P<0.001 | | |  |  |  |  |  |  |  |  |
