# Supplementary material for: Genetic Diversity of the Ralstonia solanacearum Species Complex in the Southwest Indian Ocean Islands
Source: Front Plant Sci. 2017 Dec 19;8:2139. doi: 10.3389/fpls.2017.02139 (PMC5742265; doi:10.3389/fpls.2017.02139)
Supplement: Table S4 — GenBank accession numbers for the partial sequences of seven loci used in this study. New accession numbers of sequences are in bold. [file Table4.docx]

| **RUN ID** | **Lab ID** | **Gene** | | | | | | |
| --- | --- | --- | --- | --- | --- | --- | --- | --- |
|  |  | ***gdhA*** | ***gyrB*** | ***rplB*** | ***leuS*** | ***adK*** | ***mutS*** | ***egl*** |
| RUN0001 | IPO1609 | JF702497 | JF702590 | JF702200 | JF702683 | JF702293 | EF371849 | EF371814 |
| RUN0009 | A3909 | JF702413 | JF702506 | JF702116 | JF702599 | JF702209 | AY756753 | EF371812 |
| RUN0014 | ACH732 | JF702414 | JF702507 | JF702117 | JF702600 | JF702210 | AY756743 | GQ907150 |
| RUN0016 | CFBP6784 | JF702415 | JF702508 | JF702118 | JF702601 | JF702211 | AY756742 | EF371813 |
| RUN0017 | CFBP6783 | JF702416 | JF702509 | JF702119 | JF702602 | JF702212 | EF371852 | EF371817 |
| RUN0018 | ANT80 | JF702417 | JF702510 | JF702120 | JF702603 | JF702213 | EF371854 | EF371819 |
| RUN0022 | B34 | JF702418 | JF702511 | JF702121 | JF702604 | JF702214 | JF702691 | GQ907154 |
| RUN0027 | CFBP2957 | JF702494 | JF702587 | JF702197 | JF702680 | JF702290 | EF371845 | AF295265 |
| RUN0028 | CFBP2958 | JF702419 | JF702512 | JF702122 | JF702605 | JF702215 | AY756806 | AF295266 |
| RUN0030 | CFBP2972 | JF702420 | JF702513 | JF702123 | JF702606 | JF702216 | AY756807 | AF295264 |
| RUN0039 | CFBP3059 | JF702421 | JF702514 | JF702124 | JF702607 | JF702217 | AY756766 | AF295270 |
| RUN0040 | CIP10 | JF702422 | JF702515 | JF702125 | JF702608 | JF702218 | AY756789 | AF295260 |
| RUN0041 | CIP117 | JF702423 | JF702516 | JF702126 | JF702609 | JF702219 | AY756796 | JF702301 |
| RUN0042 | CIP120 | JF702424 | JF702517 | JF702127 | JF702610 | JF702220 | AY756774 | GQ907152 |
| RUN0043 | CIP239 | JF702425 | JF702518 | JF702128 | JF702611 | JF702221 | AY756808 | AF295269 |
| RUN0044 | ACH92 | JF702426 | JF702519 | JF702129 | JF702612 | JF702222 | AY756764 | AF295254 |
| RUN0045 | CIP301 | JF702427 | JF702520 | JF702130 | JF702613 | JF702223 | JF702692 | JF702302 |
| RUN0047 | CIP365 | JF702428 | JF702521 | JF702131 | JF702614 | JF702224 | AY756787 | GQ907151 |
| RUN0054 | GMI1000 | JF702496 | JF702589 | JF702199 | JF702682 | JF702292 | AY756804 | AF295251 |
| RUN0055 | ICMP7963 | JF702429 | JF702522 | JF702132 | JF702615 | JF702225 | AY766776 | AF295263 |
| RUN0056 | J25 | JF702430 | JF702523 | JF702133 | JF702616 | JF702226 | AY756810 | AF295279 |
| RUN0060 | JT525 | JF702431 | JF702524 | JF702134 | JF702617 | JF702227 | AY756786 | AF295272 |
| RUN0061 | JT528 | KU256066 | KU255964 | KU256168 | KU256117 | KU256061 | KU255863 | KU255921 |
| RUN0062 | R229 | JF702493 | JF702586 | JF702196 | JF702679 | JF702289 | JF702728 | GU295045 |
| RUN0063 | R230 | JF702432 | JF702525 | JF702135 | JF702618 | JF702228 | AY756788 | JF702303 |
| RUN0064 | JT663 | JF702433 | JF702526 | JF702136 | JF702619 | JF702229 | JF702693 | JF702304 |
| RUN0065 | CFBP2047 | JF702434 | JF702527 | JF702137 | JF702620 | JF702230 | AY756799 | AF295262 |
| RUN0069 | MAFF211266 | JF702435 | JF702528 | JF702138 | JF702621 | JF702231 | JF702694 | AF295250 |
| RUN0071 | MAAF301558 | JF702436 | JF702529 | JF702139 | JF702622 | JF702232 | AY756752 | DQ011558 |
| RUN0074 | Molk2 | JF702498 | JF702591 | JF702201 | JF702684 | JF702294 | EF371848 | EF371841 |
| RUN0075 | NCPPB0332 | JF702437 | JF702530 | JF702140 | JF702623 | JF702233 | AY756760 | AF295276 |
| RUN0076 | NCPPB0342 | JF702438 | JF702531 | JF702141 | JF702624 | JF702234 | AY756815 | JF702305 |
| RUN0081 | NCPPB3987 | JF702439 | JF702532 | JF702142 | JF702625 | JF702235 | AY756785 | AF295261 |
| RUN0083 | PSI07 | JF702499 | JF702592 | JF702202 | JF702685 | JF702295 | JF702730 | EF371804 |
| RUN0085 | PSS190 | JF702440 | JF702533 | JF702143 | JF702626 | JF702236 | JF702695 | EU407285 |
| RUN0089 | R28 | JF702441 | JF702534 | JF702144 | JF702627 | JF702237 | AY756777 | DQ011552 |
| RUN0090 | R288 | JF702442 | JF702535 | JF702145 | JF702628 | JF702238 | AY756797 | GQ907153 |
| RUN0091 | R292 | JF702443 | JF702536 | JF702146 | JF702629 | JF702239 | AY756801 | AF295255 |
| RUN0098 | K179 | JF702444 | JF702537 | JF702147 | JF702630 | JF702240 | AY756763 | JF702306 |
| RUN0100 | UW308 | **KY862435** | **KY862576** | **KY862717** | **KY862858** | **KY862999** | **KY863140** | **KY863278** |
| RUN0104 | UW395 | **KY862436** | **KY862577** | **KY862718** | **KY862859** | **KY863000** | **KY863141** | **KY863279** |
| RUN0133 | CMR15 | JF702495 | JF702588 | JF702198 | JF702681 | JF702291 | JF702729 | KU255925 |
| RUN0145 | CFBP6942 | JF702445 | JF702538 | JF702148 | JF702631 | JF702241 | EF439773 | EF439749 |
| RUN0147 | CFBP7029 | JF702446 | JF702539 | JF702149 | JF702632 | JF702242 | EF439810 | EF439750 |
| RUN0150 | CFBP7032 | JF702447 | JF702540 | JF702150 | JF702633 | JF702243 | EF439803 | EF439726 |
| RUN0155 | PSS366 | JF702448 | JF702541 | JF702151 | JF702634 | JF702244 | JF702696 | EU407299 |
| RUN0156 | PSS216 | JF702449 | JF702542 | JF702152 | JF702635 | JF702245 | JF702697 | EU407291 |
| RUN0157 | PSS4 | JF702450 | JF702543 | JF702153 | JF702636 | JF702246 | JF702698 | EU407264 |
| RUN0159 | PSS358 | JF702451 | JF702544 | JF702154 | JF702637 | JF702247 | JF702699 | EU407298 |
| RUN0160 | JT516 | JF702452 | JF702545 | JF702155 | JF702638 | JF702248 | AY756783 | AF295258 |
| RUN0166 | CFBP7038 | JF702453 | JF702546 | JF702156 | JF702639 | JF702249 | EF439783 | EF439729 |
| RUN0203 | CFBP7054 | JF702454 | JF702547 | JF702157 | JF702640 | JF702250 | EF439800 | EF439725 |
| RUN0215 | CFBP7058 | JF702455 | JF702548 | JF702158 | JF702641 | JF702251 | EF439794 | EF439740 |
| RUN0257 | PSS219 | JF702456 | JF702549 | JF702159 | JF702642 | JF702252 | JF702700 | FJ561167 |
| RUN0258 | PSS81 | JF702457 | JF702550 | JF702160 | JF702643 | JF702253 | JF702701 | FJ561066 |
| RUN0262 | UW170 | JF702458 | JF702551 | JF702161 | JF702644 | JF702254 | JF702702 | DQ011550 |
| RUN0265 | CIP418 | JF702459 | JF702552 | JF702162 | JF702645 | JF702255 | AY756809 | GU295005 |
| RUN0288 | CFBP6779 | JF702460 | JF702553 | JF702163 | JF702646 | JF702256 | EF371872 | EF371872 |
| RUN0297 | CFBP7014 | JF702461 | JF702554 | JF702164 | JF702647 | JF702257 | EF371875 | AF371831 |
| RUN0299 | IBSBF1712 | JF702462 | JF702555 | JF702165 | JF702648 | JF702258 | EF371869 | EF371833 |
| RUN0301 | IBSBF1900 | JF702463 | JF702556 | JF702166 | JF702649 | JF702259 | EF371871 | EF371839 |
| RUN0305 | MAD-002 | **KY862437** | **KY862578** | **KY862719** | **KY862860** | **KY863001** | **KY863142** | **KY863280** |
| RUN0320 | MAD-017 | JF702464 | JF702557 | JF702167 | JF702650 | JF702260 | JF702703 | GU295040 |
| RUN0332 | MAD-029 | JF702465 | JF702558 | JF702168 | JF702651 | JF702261 | JF702704 | GU295041 |
| RUN0337 | P11 | JF702466 | JF702559 | JF702169 | JF702652 | JF702262 | JF702705 | FJ561068 |
| RUN0339 | O3 | JF702467 | JF702560 | JF702170 | JF702653 | JF702263 | JF702706 | FJ561069 |
| RUN0343 | M02 | JF702468 | JF702561 | JF702171 | JF702654 | JF702264 | JF702707 | FJ561067 |
| RUN0362 | DGBBC1138 | JF702469 | JF702562 | JF702172 | JF702655 | JF702265 | JF702708 | GU295009 |
| RUN0364 | DGBBC1227 | JF702470 | JF702563 | JF702173 | JF702656 | JF702266 | JF702709 | GU295011 |
| RUN0369 | DGBBC1125 | JF702471 | JF702564 | JF702174 | JF702657 | JF702267 | JF702710 | GU295008 |
| RUN0436 | 6035 | JF702472 | JF702565 | JF702175 | JF702658 | JF702268 | JF702711 | JF702307 |
| RUN0448 | T1-UY | JF702473 | JF702566 | JF702176 | JF702659 | JF702269 | JF702712 | GU295049 |
| RUN0449 | UW551 | JF702505 | JF702598 | JF702208 | JF702690 | JF702300 | JF702736 | DQ657596 |
| RUN0452 | CFBP1409 | JF702474 | JF702567 | JF702177 | JF702660 | JF702270 | AY756751 | EF371808 |
| RUN0454 | UW181 | JF702475 | JF702568 | JF702178 | JF702661 | JF702271 | AY756754 | GU295053 |
| RUN0471 | JT519 | JF702476 | JF702569 | JF702179 | JF702662 | JF702272 | JF702713 | GU295032 |
| RUN0477 | CFBP734 | KU256088 | KU255986 | KU256190 | KU256139 | KU256039 | KU255890 | KU255934 |
| RUN0479 | NCPPB1018 | JF702477 | JF702570 | JF702180 | JF702663 | JF702273 | AY756772 | AF295271 |
| RUN0482 | CIP240 | JF702478 | JF702571 | JF702181 | JF702664 | JF702274 | JF702714 | EF647739 |
| RUN0487 | LD74 | **KY862438** | **KY862579** | **KY862720** | **KY862861** | **KY863002** | **KY863143** | **KY863281** |
| RUN0504 | LD091 | **KY862439** | **KY862580** | **KY862721** | **KY862862** | **KY863003** | **KY863144** | **KY863282** |
| RUN0544 | RF30 | JF702479 | JF702572 | JF702182 | JF702665 | JF702275 | JF702715 | JF702308 |
| RUN0549 | RF38 | JF702480 | JF702573 | JF702183 | JF702666 | JF702276 | JF702716 | JF702309 |
| RUN0560 | RF66 | JF702481 | JF702574 | JF702184 | JF702667 | JF702277 | JF702717 | JF702310 |
| RUN0585 | GMI8044 | JF702482 | JF702575 | JF702185 | JF702668 | JF702278 | JF702718 | GU295013 |
| RUN0586 | UW163 | JF702483 | JF702576 | JF702186 | JF702669 | JF702279 | AY756779 | GU295052 |
| RUN0597 | GMI8254 | JF702484 | JF702577 | JF702187 | JF702670 | JF702280 | JF702719 | GU295014 |
| RUN0608 | JT523 | **KY862440** | **KY862581** | **KY862722** | **KY862863** | **KY863004** | AY756803 | AF295252 |
| RUN0657 | CFBP2146 | KU256091 | KU255989 | KU256193 | KU256142 | KU256036 | KU255893 | KU255938 |
| RUN0939 | JQ1118 | **KY862441** | **KY862582** | **KY862723** | **KY862864** | **KY863005** | **KY863145** | **KY863283** |
| RUN1238 | CFBP4963 | KU256093 | KU255991 | KU256195 | KU256144 | KU256034 | KU255895 | KU255940 |
| RUN1357 | UW519 | JF702485 | JF702578 | JF702188 | JF702671 | JF702281 | JF702720 | JF702311 |
| RUN1359 | UW521 | JF702486 | JF702579 | JF702189 | JF702672 | JF702282 | JF702721 | JF702312 |
| RUN1360 | R001 | JF702503 | JF702596 | JF702206 | JF702688 | JF702298 | JF702734 | JF702320 |
| RUN1361 | UW522 | JF702487 | JF702580 | JF702190 | JF702673 | JF702283 | JF702722 | JF702313 |
| RUN1431 | 07.040.3 | **KY862442** | **KY862583** | **KY862724** | **KY862865** | **KY863006** | **KY863146** | **KY863284** |
| RUN1432 | 07.040.6 | **KY862443** | **KY862584** | **KY862725** | **KY862866** | **KY863007** | **KY863147** | **KY863285** |
| RUN1439 | 07.040.21 | **KY862444** | **KY862585** | **KY862726** | **KY862867** | **KY863008** | **KY863148** | **KY863286** |
| RUN1526 | AW1 | JF702488 | JF702581 | JF702191 | JF702674 | JF702284 | JF702723 | JF702314 |
| RUN1528 | UW568 | JF702490 | JF702583 | JF702193 | JF702676 | JF702286 | JF702725 | JF702316 |
| RUN1529 | UW588 | JF702491 | JF702584 | JF702194 | JF702677 | JF702287 | JF702726 | JF702317 |
| RUN1530 | UW595 | JF702492 | JF702585 | JF702195 | JF702678 | JF702288 | JF702727 | JF702318 |
| RUN1740 | CIV19 | **KY862445** | **KY862586** | **KY862727** | **KY862868** | **KY863009** | JN798651 | JN798727 |
| RUN1930 | ech7 | **KY862446** | **KY862587** | **KY862728** | **KY862869** | **KY863010** | **KY863149** | **KY863287** |
| RUN2083 | YT11.107 | **KY862447** | **KY862588** | **KY862729** | **KY862870** | **KY863011** | **KY863150** | **KY863288** |
| RUN2127 | YT16.163 | **KY862448** | **KY862589** | **KY862730** | **KY862871** | **KY863012** | **KY863151** | **KY863289** |
| RUN2144 | YT19.185 | **KY862449** | **KY862590** | **KY862731** | **KY862872** | **KY863013** | **KY863152** | **KY863290** |
| RUN2217 | P1 | **KY862450** | **KY862591** | **KY862732** | **KY862873** | **KY863014** | **KY863153** | **KY863291** |
| RUN2267 | TG5C | **KY862451** | **KY862592** | **KY862733** | **KY862874** | **KY863015** | **KY863154** | **KY863292** |
| RUN2276 | MG6 | **KY862452** | **KY862593** | **KY862734** | **KY862875** | **KY863016** | **KY863155** | **KY863293** |
| RUN2310 | MG44 | **KY862453** | **KY862594** | **KY862735** | **KY862876** | **KY863017** | **KY863156** | **KY863294** |
| RUN2340 | MG78 | **MF448529** | **MF448530** | **MF448531** | **MF448532** | **MF448533** | **MF448534** | **MF448535** |
| RUN2344 | MG82 | **MF448536** | **MF448537** | **MF448538** | **MF448539** | **MF448540** | **MF448541** | **MF448542** |
| RUN2510 | MG275 | **KY862454** | **KY862595** | **KY862736** | **KY862877** | **KY863018** | **KY863157** | **KY863295** |
| RUN2658 | MG350 | **KY862455** | **KY862596** | **KY862737** | **KY862878** | **KY863019** | **KY863158** | **KY863296** |
| RUN2677 | MG392 | **KY862456** | **KY862597** | **KY862738** | **KY862879** | **KY863020** | **KY863159** | **KY863297** |
| RUN2706 | MG533 | **KY862457** | **KY862598** | **KY862739** | **KY862880** | **KY863021** | **KY863160** | **KY863298** |
| RUN2717 | MG546 | **KY862458** | **KY862599** | **KY862740** | **KY862881** | **KY863022** | **KY863161** | **KY863299** |
| RUN2722 | MG551 | **KY862459** | **KY862600** | **KY862741** | **KY862882** | **KY863023** | **KY863162** | **KY863300** |
| RUN2735 | MG564 | **KY862460** | **KY862601** | **KY862742** | **KY862883** | **KY863024** | **KY863163** | **KY863301** |
| RUN2786 | MG621 | **KY862461** | **KY862602** | **KY862743** | **KY862884** | **KY863025** | **KY863164** | **KY863302** |
| RUN2996 | P6 | **KY862462** | **KY862603** | **KY862744** | **KY862885** | **KY863026** | **KY863165** | **KY863303** |
| RUN3083 | 86 | **KY862463** | **KY862604** | **KY862745** | **KY862886** | **KY863027** | **KY863166** | **KY863304** |
| RUN3148 | 166 | **KY862464** | **KY862605** | **KY862746** | **KY862887** | **KY863028** | **KY863167** | **KY863305** |
| RUN3216 | 257 | **KY862465** | **KY862606** | **KY862747** | **KY862888** | **KY863029** | **KY863168** | **KY863306** |
| RUN3230 | 272 | **KY862466** | **KY862607** | **KY862748** | **KY862889** | **KY863030** | **KY863169** | **KY863307** |
| RUN3277 | 368 | **KY862467** | **KY862608** | **KY862749** | **KY862890** | **KY863031** | **KY863170** | **KY863308** |
| RUN3304 | 410 | **KY862468** | **KY862609** | **KY862750** | **KY862891** | **KY863032** | **KY863171** | **KY863309** |
| RUN3321 | 434 | **KY862469** | **KY862610** | **KY862751** | **KY862892** | **KY863033** | **KY863172** | **KY863310** |
| RUN3489 | LB107 | **KY862470** | **KY862611** | **KY862752** | **KY862893** | **KY863034** | **KY863173** | **KY863311** |
| RUN3490 | JT675 | **KY862471** | **KY862612** | **KY862753** | **KY862894** | **KY863035** | **KY863174** | **KY863312** |
| RUN3491 | LB217 | **KY862472** | **KY862613** | **KY862754** | **KY862895** | **KY863036** | **KY863175** | **KY863313** |
| RUN3492 | LB218 | **KY862473** | **KY862614** | **KY862755** | **KY862896** | **KY863037** | **KY863176** | **KY863314** |
| RUN3493 | LB219 | **KY862474** | **KY862615** | **KY862756** | **KY862897** | **KY863038** | **KY863177** | **KY863315** |
| RUN3494 | LB220 | **KY862475** | **KY862616** | **KY862757** | **KY862898** | **KY863039** | **KY863178** | **KY863316** |
| RUN3495 | LB221 | **KY862476** | **KY862617** | **KY862758** | **KY862899** | **KY863040** | **KY863179** | **KY863317** |
| RUN3501 | JS940 | **KY862477** | **KY862618** | **KY862759** | **KY862900** | **KY863041** | **KY863180** | **KY863318** |
| RUN3503 | 66 | **KY862478** | **KY862619** | **KY862760** | **KY862901** | **KY863042** | **KY863181** | **KY863319** |
| RUN3505 | JS944 | **KY862479** | **KY862620** | **KY862761** | **KY862902** | **KY863043** | **KY863182** | **KY863320** |
| RUN3510 | JS954 | **KY862480** | **KY862621** | **KY862762** | **KY862903** | **KY863044** | **KY863183** | **KY863321** |
| RUN3513 | JS955 | **KY862481** | **KY862622** | **KY862763** | **KY862904** | **KY863045** | **KY863184** | **KY863322** |
| RUN3560 | Rs-TH-BG-1 | **KY862482** | **KY862623** | **KY862764** | **KY862905** | **KY863046** | **KY863185** | **KY863323** |
| RUN3563 | Rs-TH-EGP-2 | **KY862483** | **KY862624** | **KY862765** | **KY862906** | **KY863047** | **KY863186** | **KY863324** |
| RUN3564 | Rs-TH-MA-1 | **KY862484** | **KY862625** | **KY862766** | **KY862907** | **KY863048** | **KY863187** | **KY863325** |
| RUN3573 | BMa-D16 | **KY862485** | **KY862626** | **KY862767** | **KY862908** | **KY863049** | **KY863188** | **KY863326** |
| RUN3611 | PR1.4b(1) | **KY862486** | **KY862627** | **KY862768** | **KY862909** | **KY863050** | **KY863189** | **KY863327** |
| RUN3633 | 42796 | **KY862487** | **KY862628** | **KY862769** | **KY862910** | **KY863051** | KP730686 | KP730640 |
| RUN3666 | T2 | **KY862488** | **KY862629** | **KY862770** | **KY862911** | **KY863052** | **KY863190** | **KY863328** |
| RUN3691 | Chamand | **KY862489** | **KY862630** | **KY862771** | **KY862912** | **KY863053** | **KY863191** | **KY863329** |
| RUN3699 | Dom 8.7 | **KY862490** | **KY862631** | **KY862772** | **KY862913** | **KY863054** | **KY863192** | **KY863330** |
| RUN3711 | 2,1 | **KY862491** | **KY862632** | **KY862773** | **KY862914** | **KY863055** | **KY863193** | **KY863331** |
| RUN3714 | N'1 (7) | **KY862492** | **KY862633** | **KY862774** | **KY862915** | **KY863056** | **KY863194** | **KY863332** |
| RUN3732 | 6,1 | **KY862493** | **KY862634** | **KY862775** | **KY862916** | **KY863057** | **KY863195** | **KY863333** |
| RUN3733 | 6,2 | **KY862494** | **KY862635** | **KY862776** | **KY862917** | **KY863058** | **KY863196** | **KY863334** |
| RUN3740 | 8,1 | **KY862495** | **KY862636** | **KY862777** | **KY862918** | **KY863059** | **KY863197** | **KY863335** |
| RUN3743 | Tony 36 | **KY862496** | **KY862637** | **KY862778** | **KY862919** | **KY863060** | **KY863198** | **KY863336** |
| RUN3744 | 9,2 | **KY862497** | **KY862638** | **KY862779** | **KY862920** | **KY863061** | **KY863199** | **KY863337** |
| RUN3756 | Bruno1 | **KY862498** | **KY862639** | **KY862780** | **KY862921** | **KY863062** | **KY863200** | **KY863338** |
| RUN3770 | RAS1 | **KY862499** | **KY862640** | **KY862781** | **KY862922** | **KY863063** | **KY863201** | **KY863339** |
| RUN3812 | S1T1 | **KY862500** | **KY862641** | **KY862782** | **KY862923** | **KY863064** | **KY863202** | **KY863340** |
| RUN3815 | JCB3 | **KY862501** | **KY862642** | **KY862783** | **KY862924** | **KY863065** | **KY863203** | **KY863341** |
| RUN3841 | O1 | **KY862502** | **KY862643** | **KY862784** | **KY862925** | **KY863066** | **KY863204** | **KY863342** |
| RUN3856 | DR1G | **KY862503** | **KY862644** | **KY862785** | **KY862926** | **KY863067** | **KY863205** | **KY863343** |
| RUN3862 | SG14 | **KY862504** | **KY862645** | **KY862786** | **KY862927** | **KY863068** | **KY863206** | **KY863344** |
| RUN3869 | SG14 | **KY862505** | **KY862646** | **KY862787** | **KY862928** | **KY863069** | **KY863207** | **KY863345** |
| RUN3884 | EG13 | **KY862506** | **KY862647** | **KY862788** | **KY862929** | **KY863070** | **KY863208** | **KY863346** |
| RUN3893 | LJCT2 | **KY862507** | **KY862648** | **KY862789** | **KY862930** | **KY863071** | **KY863209** | **KY863347** |
| RUN3928 | EGCG1 | **KY862508** | **KY862649** | **KY862790** | **KY862931** | **KY863072** | **KY863210** | **KY863348** |
| RUN4047 | HGT1 | **KY862509** | **KY862650** | **KY862791** | **KY862932** | **KY863073** | **KY863211** | **KY863349** |
| RUN4076 | MFbrede1 | **KY862510** | **KY862651** | **KY862792** | **KY862933** | **KY863074** | **KY863212** | **KY863350** |
| RUN4079 | MFT8 | **KY862511** | **KY862652** | **KY862793** | **KY862934** | **KY863075** | **KY863213** | **KY863351** |
| RUN4082 | GJBG4 | **KY862512** | **KY862653** | **KY862794** | **KY862935** | **KY863076** | **KY863214** | **KY863352** |
| RUN4105 | HRGP1 | **KY862513** | **KY862654** | **KY862795** | **KY862936** | **KY863077** | **KY863215** | **KY863353** |
| RUN4112 | HRA1 | **KY862514** | **KY862655** | **KY862796** | **KY862937** | **KY863078** | **KY863216** | **KY863354** |
| RUN4170 | MNT1 | **KY862515** | **KY862656** | **KY862797** | **KY862938** | **KY863079** | **KY863217** | **KY863355** |
| RUN4196 | MF far 1 | **KY862516** | **KY862657** | **KY862798** | **KY862939** | **KY863080** | **KY863218** | **KY863356** |
| RUN4210 | RE 1.3.2 | **KY862517** | **KY862658** | **KY862799** | **KY862940** | **KY863081** | **KY863219** | **KY863357** |
| RUN4257 | Haricot1 | **KY862518** | **KY862659** | **KY862800** | **KY862941** | **KY863082** | **KY863220** | **KY863358** |
| RUN4263 | Adv2 | **KY862519** | **KY862660** | **KY862801** | **KY862942** | **KY863083** | **KY863221** | **KY863359** |
| RUN4292 | 44 | **KY862520** | **KY862661** | **KY862802** | **KY862943** | **KY863084** | **KY863222** | **KY863360** |
| RUN4303 | 122 | **KY862521** | **KY862662** | **KY862803** | **KY862944** | **KY863085** | **KY863223** | **KY863361** |
| RUN4308 | 145 | **KY862522** | **KY862663** | **KY862804** | **KY862945** | **KY863086** | **KY863224** | **KY863362** |
| RUN4311 | 172 | **KY862523** | **KY862664** | **KY862805** | **KY862946** | **KY863087** | **KY863225** | **KY863363** |
| RUN4315 | 214 | **KY862524** | **KY862665** | **KY862806** | **KY862947** | **KY863088** | **KY863226** | **KY863364** |
| RUN4325 | 240 | **KY862525** | **KY862666** | **KY862807** | **KY862948** | **KY863089** | **KY863227** | **KY863365** |
| RUN4326 | 241 | **KY862526** | **KY862667** | **KY862808** | **KY862949** | **KY863090** | **KY863228** | **KY863366** |
| RUN4337 | 87 | **KY862527** | **KY862668** | **KY862809** | **KY862950** | **KY863091** | **KY863229** | **KY863367** |
| RUN4376 | 3 | **KY862528** | **KY862669** | **KY862810** | **KY862951** | **KY863092** | **KY863230** | **KY863368** |
| RUN4392 | 43 | **KY862529** | **KY862670** | **KY862811** | **KY862952** | **KY863093** | **KY863231** | **KY863369** |
| RUN4393 | 46 K | **KY862530** | **KY862671** | **KY862812** | **KY862953** | **KY863094** | **KY863232** | **KY863370** |
| RUN4400 | 83 | **KY862531** | **KY862672** | **KY862813** | **KY862954** | **KY863095** | **KY863233** | **KY863371** |
| RUN4406 | 89 amaranthe | **KY862532** | **KY862673** | **KY862814** | **KY862955** | **KY863096** | **KY863234** | **KY863372** |
| RUN4407 | 90 | **KY862533** | **KY862674** | **KY862815** | **KY862956** | **KY863097** | **KY863235** | **KY863373** |
| RUN4438 | Pepino 7 | **KY862534** | **KY862675** | **KY862816** | **KY862957** | **KY863098** | **KY863236** | **KY863374** |
| RUN4450 | GP1 | **KY862535** | **KY862676** | **KY862817** | **KY862958** | **KY863099** | **KY863237** | **KY863375** |
| RUN4478 | BM3 | **KY862536** | **KY862677** | **KY862818** | **KY862959** | **KY863100** | **KY863238** | **KY863376** |
| RUN4509 | Tu2P3 | **KY862537** | **KY862678** | **KY862819** | **KY862960** | **KY863101** | **KY863239** | **KY863377** |
| RUN4584 | 1,3 | **KY862538** | **KY862679** | **KY862820** | **KY862961** | **KY863102** | **KY863240** | **KY863378** |
| RUN4603 | 2,1 | **KY862539** | **KY862680** | **KY862821** | **KY862962** | **KY863103** | **KY863241** | **KY863379** |
| RUN4606 | 2,4 | **KY862540** | **KY862681** | **KY862822** | **KY862963** | **KY863104** | **KY863242** | **KY863380** |
| RUN4645 | 3,4 | **KY862541** | **KY862682** | **KY862823** | **KY862964** | **KY863105** | **KY863243** | **KY863381** |
| RUN4664 | 4,29 | **KY862542** | **KY862683** | **KY862824** | **KY862965** | **KY863106** | **KY863244** | **KY863382** |
| RUN4680 | 11,1 | **KY862543** | **KY862684** | **KY862825** | **KY862966** | **KY863107** | **KY863245** | **KY863383** |
| RUN4847 | 2,7 | **KY862544** | **KY862685** | **KY862826** | **KY862967** | **KY863108** | **KY863246** | **KY863384** |
| RUN4899 | 20,1 | **KY862545** | **KY862686** | **KY862827** | **KY862968** | **KY863109** | **KY863247** | **KY863385** |
| RUN4953 | 22,2 | **KY862546** | **KY862687** | **KY862828** | **KY862969** | **KY863110** | **KY863248** | **KY863386** |
| RUN5007 | 23,1 | **KY862547** | **KY862688** | **KY862829** | **KY862970** | **KY863111** | **KY863249** | **KY863387** |
| RUN5135 | 25,1 | **KY862548** | **KY862689** | **KY862830** | **KY862971** | **KY863112** | **KY863250** | **KY863388** |
| RUN5137 | 8,2 | **KY862549** | **KY862690** | **KY862831** | **KY862972** | **KY863113** | **KY863251** | **KY863389** |
| RUN5138 | 6,27 | **KY862550** | **KY862691** | **KY862832** | **KY862973** | **KY863114** | **KY863252** | **KY863390** |
| RUN5163 | 19,3 | **KY862551** | **KY862692** | **KY862833** | **KY862974** | **KY863115** | **KY863253** | **KY863391** |
| RUN5253 | 1.31T | **KY862552** | **KY862693** | **KY862834** | **KY862975** | **KY863116** | **KY863254** | **KY863392** |
| RUN5274 | 2,32 | **KY862553** | **KY862694** | **KY862835** | **KY862976** | **KY863117** | **KY863255** | **KY863393** |
| RUN5277 | 2,28 | **KY862554** | **KY862695** | **KY862836** | **KY862977** | **KY863118** | **KY863256** | **KY863394** |
| RUN5331 | M3H1.1 | **KY862555** | **KY862696** | **KY862837** | **KY862978** | **KY863119** | **KY863257** | **KY863395** |
| RUN5385 | M3A7.63 | **KY862556** | **KY862697** | **KY862838** | **KY862979** | **KY863120** | **KY863258** | **KY863396** |
| RUN5419 | M3T10.127 | **KY862557** | **KY862698** | **KY862839** | **KY862980** | **KY863121** | **KY863259** | **KY863397** |
| RUN5445 | M3Pi9.107 | **KY862558** | **KY862699** | **KY862840** | **KY862981** | **KY863122** | **KY863260** | **KY863398** |
| RUN5453 | M3T4.59 | **KY862559** | **KY862700** | **KY862841** | **KY862982** | **KY863123** | **KY863261** | **KY863399** |
| RUN5454 | M3An5.60 | **KY862560** | **KY862701** | **KY862842** | **KY862983** | **KY863124** | **KY863262** | **KY863400** |
| RUN5455 | M3An6.61 | **KY862561** | **KY862702** | **KY862843** | **KY862984** | **KY863125** | **KY863263** | **KY863401** |
| RUN5456 | M3Pi16.175 | **KY862562** | **KY862703** | **KY862844** | **KY862985** | **KY863126** | **KY863264** | **KY863402** |
| RUN5479 | BDMT1 | **KY862563** | **KY862704** | **KY862845** | **KY862986** | **KY863127** | **KY863265** | **KY863403** |
| RUN5480 | BA2-2-PT3 | **KY862564** | **KY862705** | **KY862846** | **KY862987** | **KY863128** | **KY863266** | **KY863404** |
| RUN5481 | KOD2A2 | **KY862565** | **KY862706** | **KY862847** | **KY862988** | **KY863129** | **KY863267** | **KY863405** |
| RUN5482 | NDRB2T1 | **KY862566** | **KY862707** | **KY862848** | **KY862989** | **KY863130** | **KY863268** | **KY863406** |
| RUN5485 | NIM3T1 | **KY862567** | **KY862708** | **KY862849** | **KY862990** | **KY863131** | **KY863269** | **KY863407** |
| RUN5489 | WAL2T1 | **KY862568** | **KY862709** | **KY862850** | **KY862991** | **KY863132** | **KY863270** | **KY863408** |
| RUN5495 | NIM1T1 | **KY862569** | **KY862710** | **KY862851** | **KY862992** | **KY863133** | **KY863271** | **KY863409** |
| RUN5508 | NDRB1T1 | **KY862570** | **KY862711** | **KY862852** | **KY862993** | **KY863134** | **KY863272** | **KY863410** |
| RUN5513 | LINT1 | **KY862571** | **KY862712** | **KY862853** | **KY862994** | **KY863135** | **KY863273** | **KY863411** |
| RUN5514 | NIM2T1 | **KY862572** | **KY862713** | **KY862854** | **KY862995** | **KY863136** | **KY863274** | **KY863412** |
| RUN5516 | MRMA1 | **KY862573** | **KY862714** | **KY862855** | **KY862996** | **KY863137** | **KY863275** | **KY863413** |
| RUN5521 | OUAT1 | **KY862574** | **KY862715** | **KY862856** | **KY862997** | **KY863138** | **KY863276** | **KY863414** |
| RUN5525 | PATT3 | **KY862575** | **KY862716** | **KY862857** | **KY862998** | **KY863139** | **KY863277** | **KY863415** |
