# Supplementary material for: Genetic Diversity of the Ralstonia solanacearum Species Complex in the Southwest Indian Ocean Islands
Source: Front Plant Sci. 2017 Dec 19;8:2139. doi: 10.3389/fpls.2017.02139 (PMC5742265; doi:10.3389/fpls.2017.02139)
Supplement: Table S5 — Substitution models of the seven genes studied using Jmodeltest 2. [file Table5.docx]

| **Gene** | **Substitution model** |
| --- | --- |
| ***gdhA*** | Tamura-Nei^1^ (TrN) + G* |
| ***gyrB*** | Hasegawa-Kishino-Yano^2^ (HKY) + I* + G |
| ***rplB*** | Tamura-Nei (TrN) + G |
| ***leuS*** | Tamura-Nei (TrN) + I |
| ***adk*** | Hasegawa-Kishino-Yano (HKY) + I |
| ***mutS*** | Hasegawa-Kishino-Yano (HKY) + I + G |
| ***egl*** | Tamura-Nei (TrN) + G |
| **Concatenated sequences** | Transitional model^3^ (TIM1) + I + G |

* G = Gamma distribution

* I =Proportion of invariable sites

^1^ Variable base frequencies, equal transversion rates, variable transition rates ([Tamura Nei 1993](http://www.ncbi.nlm.nih.gov/pubmed/8336541))

^2^ Variable base frequencies, one transition rate and one transversion rate (Hasegawa *et al.,* 1985)

^3^ Transitional model, variable base frequencies, variable transition rates, two transversion rates (Posada 2003)
