# Supplementary material for: Genetic Diversity of the Ralstonia solanacearum Species Complex in the Southwest Indian Ocean Islands
Source: Front Plant Sci. 2017 Dec 19;8:2139. doi: 10.3389/fpls.2017.02139 (PMC5742265; doi:10.3389/fpls.2017.02139)
Supplement: Table S6 — Comparison between the seven genes (gdhA, gyrB, rplB, leuS, adk, mutS, and egl) and the concatenated-genes trees generated by the congruency Index Icong values. Icong, p-value, and MAST were indicated in this table. [file Table6.DOCX]

| **Loci** | ***gdhA*** | | | ***gyrB*** | | | ***rplB*** | | | ***leuS*** | | | ***adk*** | | | ***mutS*** | | | ***egl*** | | | **Concatenate** | | |
| --- | --- | --- | --- | --- | --- | --- | --- | --- | --- | --- | --- | --- | --- | --- | --- | --- | --- | --- | --- | --- | --- | --- | --- | --- |
|  | I cong | p-value | MAST | I cong | p-value | MAST^a^ | I cong | p-value | MAST | I cong | p-value | MAST | I cong | p-value | MAST | I cong | p-value | MAST | I cong | p-value | MAST | I cong | p-value | MAST |
| ***gdhA*** |  |  |  | 5.005 | 5.69E-53 | 113 | 6.599 | 7.56E-74 | 149 | 6.954 | 1.73E-78 | 157 | 4.872 | 3.13E-51 | 110 | 5.846 | 5.46E-64 | 132 | 4.385 | 7.48E-45 | 99 | 3.278 | 2.36E-30 | 74 |
| ***gyrB*** |  |  |  |  |  |  | 5.403 | 3.44E-58 | 122 | 5.403 | 3.44E-58 | 122 | 5.581 | 1.65E-60 | 126 | 6.024 | 2.61E-66 | 136 | 3.853 | 6.81E-38 | 87 | 2.967 | 2.70E-26 | 67 |
| ***rplB*** |  |  |  |  |  |  |  |  |  | 7.441 | 7.24E-85 | 168 | 5.271 | 1.89E-56 | 119 | 6.201 | 1.25E-68 | 140 | 5.005 | 5.69E-53 | 113 | 3.366 | 1.63E-31 | 76 |
| ***leuS*** |  |  |  |  |  |  |  |  |  |  |  |  | 5.093 | 3.94E-54 | 115 | 6.068 | 6.88E-67 | 137 | 4.606 | 9.43E-48 | 104 | 3.809 | 2.59E-37 | 86 |
| ***adk*** |  |  |  |  |  |  |  |  |  |  |  |  |  |  |  | 5.536 | 6.26E-60 | 125 | 3.543 | 7.81E-34 | 80 | 2.923 | 1.03E-25 | 66 |
| ***mutS*** |  |  |  |  |  |  |  |  |  |  |  |  |  |  |  |  |  |  | 4.695 | 6.53E-49 | 106 | 3.366 | 1.63E-31 | 76 |
| ***egl*** |  |  |  |  |  |  |  |  |  |  |  |  |  |  |  |  |  |  |  |  |  | 3.278 | 2.36E-30 | 74 |
| **Concatenate** |  |  |  |  |  |  |  |  |  |  |  |  |  |  |  |  |  |  |  |  |  |  |  |  |
|  |  |  |  |  |  |  |  |  |  |  |  |  |  |  |  |  |  |  |  |  |  |  |  |  |
| *^a^* | MAST: The Maximum Agreement SubTree | | | | |  |  |  |  |  |  |  |  |  |  |  |  |  |  |  |  |  |  |  |
